# Activation of the Nuclear Factor E2-Related Factor 2 Pathway by Novel Natural Products Halomadurones A–D and a Synthetic Analogue

**DOI:** 10.3390/md11125089

**Published:** 2013-12-16

**Authors:** Thomas P. Wyche, Miranda Standiford, Yanpeng Hou, Doug Braun, Delinda A. Johnson, Jeffrey A. Johnson, Tim S. Bugni

**Affiliations:** Pharmaceutical Sciences Division, University of Wisconsin-Madison, 777 Highland Avenue, Madison, WI 53705, USA; E-Mails: twyche@wisc.edu (T.P.W.); tstandiford@wisc.edu (M.S.); yanpenghou@gmail.com (Y.H.); drbraun1@facstaff.wisc.edu (D.B.); dajohnson@pharmacy.wisc.edu (D.A.J.); jajohnson@pharmacy.wisc.edu (J.A.J.)

**Keywords:** *Ecteinascidia turbinata*, halomadurones, *Actinomadura*, nuclear factor E2-related factor antioxidant response element

## Abstract

Two novel chlorinated pyrones, halomadurones A and B, and two novel brominated analogues, halomadurones C and D, were isolated from a marine *Actinomadura* sp. cultivated from the ascidian *Ecteinascidia turbinata*. Additionally, a non-halogenated analogue, 2-methyl-6-((*E*)-3-methyl-1,3-hexadiene)-γ-pyrone, was synthesized to understand the role of the halogens for activity. Halomadurones C and D demonstrated potent nuclear factor E2-related factor antioxidant response element (Nrf2-ARE) activation, which is an important therapeutic approach for treatment of neurodegenerative diseases.

## 1. Introduction

The prevalence of neurodegenerative diseases, such as Alzheimer’s disease (AD), Parkinson’s disease, Huntington’s disease, and amyotrophic lateral sclerosis (ALS) is on the rise worldwide [1,2,3]; cases of AD, the most common neurodegenerative disease, are projected to reach 115 million by the year 2050 [3]. Meanwhile, only a handful of therapeutics are available that target these diseases, most of which only treat the symptoms not the underlying cause. Many neurodegenerative diseases are caused by mitochondrial DNA mutation or oxidative stress damage [4]. Therefore, determining methods of minimizing oxidative stress is a potential strategy towards preventing many neurodegenerative diseases [5,6].

The transcription factor Nrf2 (nuclear factor E2-related factor) activates the antioxidant response element (ARE), which is located in the promoter region of genes that encode cytoprotective and antioxidant enzymes including many phase II detoxification enzymes [7,8,9]. Therefore, activation of Nrf2 represents a promising therapeutic strategy for combatting neurodegenerative diseases. In order to identify activators of this pathway in conjunction with having an *in vivo* model for evaluating therapeutics for neurodegenerative diseases, a transgenic mouse model was created that contained a human placental alkaline phosphatase reporter under the control of the ARE. For *in vitro* testing, primary neuronal cells from ARE-hPAP transgenic reporter were used to test for Nrf2/ARE activation [10,11]. The use of primary neuronal cells has provided an *in vitro* model that most closely resembles the *in vivo* pathology, albeit without the blood brain barrier. In parallel, the 3-(4,5-dimethylthiazol-2-yl)-5-(3-carboxymethoxyphenyl)-2-(4-sulfophenyl)-2*H*-tetrazolium inner salt (MTS) assay can be used to test toxicity of the compounds. There have been only limited reports on natural product pharmacophores that activate the Nrf2/ARE pathway. The vast majority have been electrophiles, such as Michael acceptors [12,13,14,15,16]. More recently, activation of the Nrf2-ARE pathway with small molecules was also investigated by Luesch and co-workers leading to the discovery of Nrf2-ARE activators from seaweed extracts [17] and an electrophile ARE activator with *in vivo* activity [16].

**Figure 1:**
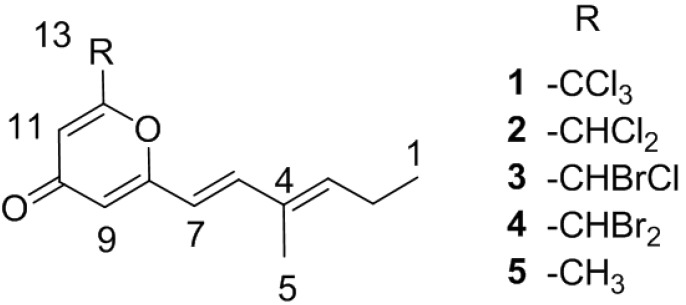
Structures of **1**–**5**.

In our pursuit of Nrf2/ARE activators, we isolated four novel halogenated electrophilic polyketides that were named halomadurones A–D (**1**–**4**) (Figure 1), from a marine *Actinomadura* sp. (Strain WMMB499) cultivated from the ascidian *Ecteinascidia turbinata* (Herdman, 1880). Halomadurones C (**3**) and D (**4**) demonstrated potent nuclear factor E2-related factor antioxidant response element (Nrf2-ARE) activation in the ARE-hPAP assay. The cytotoxicity studies and activity in the ARE-hPAP assay was suggestive of a narrow therapeutic window. Synthesis of a non-halogenated pyrone demonstrated that bromination was a key feature of the pharmacophore.

In addition to the promising *in vitro* activity, halomadurones A–D (**1**–**4**) represent a new carbon skeleton due to the position of the methyl group. Additionally, halomadurone A (**1**), contains a trichloromethyl group, which is a rare moiety in natural products. Most natural products that contain a trichloromethyl group, such as barbamide A [18] and dysidenamide [19], were isolated from cyanobacteria and also contain a thiazole ring. However, cytotoxic trichlorinated polyenones neocarzilin A and B [20] were produced by the soil-derived bacterium *Streptomyces carzinostaticus* and do not contain a thiazole ring. Consequently, neocarzilins A and B, which have been the target for various synthetic [21] and biosynthetic studies [22], are now joined by halomadurone A (**1**) as the sole members of actinomycete-derived trichlorinated natural products. The role of the trichloromethyl group in bioactivity was investigated with halomadurones A–D (**1**–**4**) and a non-halogenated analogue, 2-methyl-6-((*E*)-3-methyl-1,3-hexadiene)-γ-pyrone (5), which we synthesized. 

## 2. Results and Discussion

### 2.1. Bacterial Strain Selection and Structure Elucidation

Our attention was drawn to strain WMMB499 after analysis of thirty-four marine-derived bacterial extracts using LCMS-based metabolomics relying on principal component analysis (PCA) [23]. When compared to the other 33 LCMS chromatograms using PCA, WMMB499 was identified as having unique chemical signatures and putative novel natural products; after saline fermentation and isolation, WMMB499 was found to produce halomadurones A (**1**) and B (**2**). Halomadurones C (**3**) and D (**4**) were produced by WMMB499 by increasing the ratio KBr/NaCl. Previous studies have demonstrated that many halogenases are promiscuous with respect to using Br and Cl [24,25].

HRMS supported the molecular formula of C_13_H_13_Cl_3_O_2_ and C_13_H_14_Cl_2_O_2_ for halomadurone A (**1**) and B (**2**), respectively. Analysis of ^1^H and ^13^C NMR data (Table 1, Figure S1–S12) allowed for determination of the structures of halomadurones A (**1**) and B (**2**). The ^1^H and ^13^C NMR data for halomadurone A (**1**) supported the conclusion that C-13 was connected to only one carbon, and in combination with the ^13^C chemical shift at C-13 (90.3 ppm) and molecular formula, allowed for the assignment of the trichloromethyl group. A large vicinal coupling constant (^3^*J*_H_ 15.6) for H-6 and H-7 supported the assignment of the *E* conformation [26]. ROESY NMR data allowed for the assignment of the *E* olefin at C-3 and C-4 (Figure S13). A comparison of ^1^H and ^13^C NMR shifts between halomadurones A (**1**) and B (**2**) showed that the only difference between the two structures was at C-13. C-13 in halomadurone B (**2**) contained two chlorine atoms and one hydrogen as evidenced by the upfield shift of C-13 to 65.8 ppm and a methine proton at 6.35 ppm. The relative configuration of the two olefins was assigned the same as halomadurone A (**1**) on the basis of vicinal coupling constants and ROESY NMR data.

After the structural elucidation of halomadurones A (**1**) and B (**2**), the amount of KBr was increased from 0.1 g/L to 10 g/L and NaCl was reduced from 20 g/L to 0 g/L in fermentation medium ASW-A, resulting in the production of brominated analogues. Many halogenases have low specificity for the halide substrate, and the incorporation of a specific halogen often depends on the relative concentration of each halogen anion in the fermentation medium [27,28]. HRMS of the two brominated analogues, halomadurones C (**3**) and D (**4**), supported the molecular formulas of C_13_H_14_BrClO_2_ and C_13_H_14_Br_2_O_2_, respectively. A comparison of the ^1^H and ^13^C NMR shifts (Table 1, Figure S14–S25) of halomadurones C (**3**) and D (**4**) with halomadurone B (**2**) confirmed that the only difference in the structures was the halogenated atoms at C-13. The relative configuration of the two olefins for halomadurones C (**3**) and D (**4**) was assigned the same as halomadurone A (**1**) on the basis of vicinal coupling constants and ROESY NMR data. The lack of optical rotation by halomadurone C (**3**) suggested that a racemic mixture of both enantiomers existed.

**Table 1:** ^1^H and ^13^C NMR data for **1**–**4** (600 MHz for ^1^H, 150 MHz for ^13^C, CDCl_3_).

No.	1		2		3		4	
	δ_C_, mult.	δ_H_ (*J* in Hz)	δ_C_, mult.	δ_H_ (*J* in Hz)	δ_C_, mult.	δ_H_ (*J* in Hz)	δ_C_, mult.	δ_H_ (*J* in Hz)
1	13.8, CH_3_	1.04, t (7.3)	13.8, CH_3_	1.04, t (7.3)	13.8, CH_3_	1.04, t (7.5)	13.8, CH_3_	1.05, t (7.5)
2	22.4, CH_2_	2.24, qn (7.3)	22.4, CH_2_	2.24, qn (7.3)	22.4, CH_2_	2.24, qn (7.5)	22.5, CH_2_	2.24, qn (7.5)
3	144.3, CH	5.93, t (7.3)	144.0, CH	5.92, t (7.3)	144.0, CH	5.93, t (7.2)	144.0, CH	5.94, t (7.5)
4	132.6, C		132.6, C		132.7, C		132.7, C	
5	12.2, CH_3_	1.82, s	12.2, CH_3_	1.82, s	12.2, CH_3_	1.82, s	12.2, CH_3_	1.82, s
6	143.3, CH	7.14, d (15.6)	142.9, CH	7.14, d (15.6)	142.9, CH	7.14, d (15.8)	142.9, CH	7.17, d (15.8)
7	115.8, CH	6.07, d (15.6)	116.1, CH	6.07, d (15.6)	116.2, CH	6.04, d (15.8)	116.3, CH	6.05, d (15.8)
8	163.2, C		162.9, C		163.4, C		163.4, C	
9	112.3, CH	6.17, d (1.8)	112.7, CH	6.17, d (2.0)	112.2, CH	6.14, d (2.3)	113.1, CH	6.13, d (2.3)
10	179.5, C		179.5, C		179.6, C		179.5, C	
11	111.8, CH	6.85, d (1.8)	112.7, CH	6.42, d (2.0)	112.1, CH	6.39, d (2.3)	111.4, CH	6.32, d (2.3)
12	160.5, C		160.2, C		160.7, C		160.8, C	
13	90.3, C		65.8, CH	6.35, s	50.1, CH	6.35, s	32.5, CH	6.24, s

### 2.2. Synthetic Analogue

**Scheme 1:**
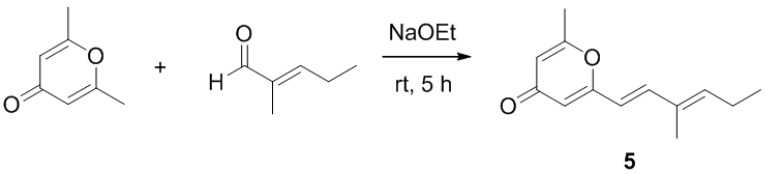
Synthesis of **5**.

After isolation and structure elucidation of halomadurones A–D (**1**–**4**), a non-halogenated analogue, 2-methyl-6-((*E*)-3-methyl-1,3-hexadiene)-γ-pyrone (**5**) was synthesized to compare the bioactivity of the novel structures (Scheme 1). In particular, the effect of substitution on the pyrone methyl was investigated. 2-6-Dimethyl-γ-pyrone in sodium ethoxide was added to 2-methyl-2-pentenal and stirred at room temperature for 5 hours [29]. Purification of the product (see Experimental Section) resulted in 4.0 mg 2-methyl-6-((*E*)-3-methyl-1,3-hexadiene)-γ-pyrone (**5**). The structure was confirmed by analysis of ^1^H and ^13^C NMR and MS data (Table S1, Figure S26–S31). ROESY NMR data and vicinal coupling constants confirmed the assignment of the *E* olefin at C-3 and C-4. Although sufficient compound yield was achieved for initial *in vitro* studies, the overall yield was low (~2%) as expected.

### 2.3. Biological Activity

**Figure 2:**
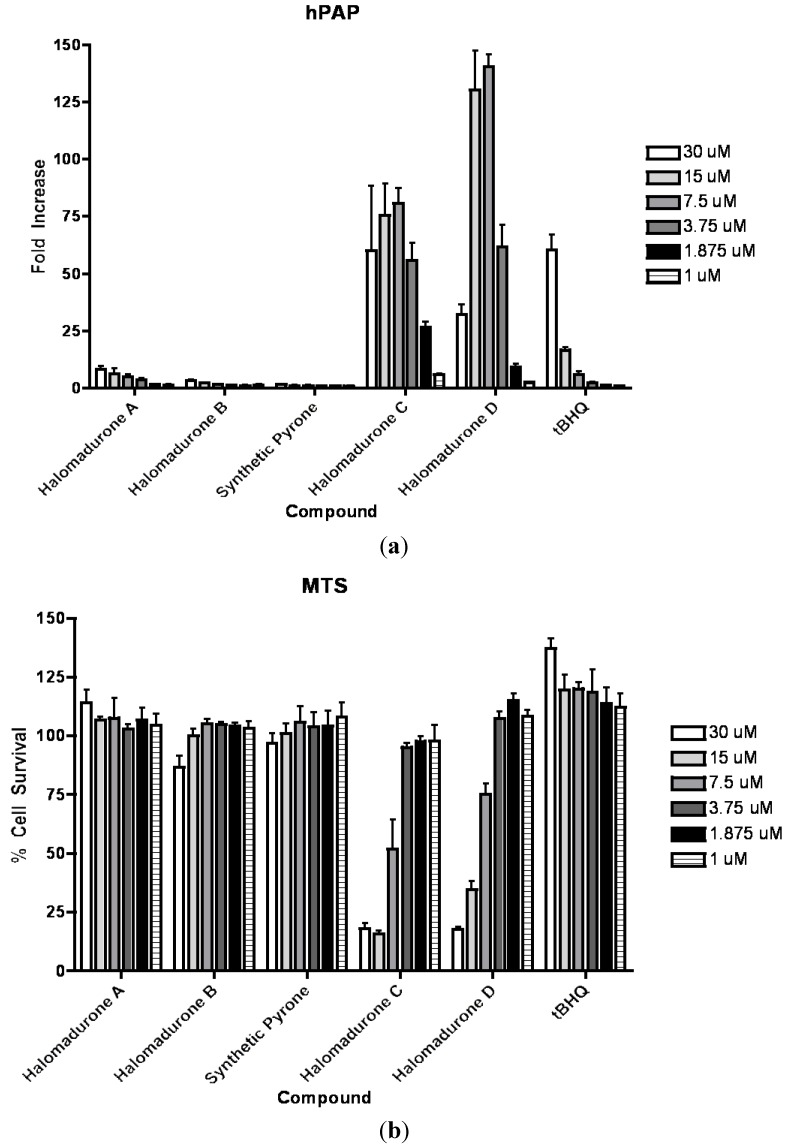
Nrf2 activation and cytotoxicity of **1**–**5**. Primary cortical cultures were prepared and treated with vehicle or compounds at increasing concentrations for 48 h. (**a**) hPAP activity was measured. All values are standardized to the vehicle treated value and presented as fold change; Mean ± SEM. (**b**) Cell viability was assessed using MTS assay. All values are standardized to the vehicle treated value and presented as percent of Mean ± SEM.

On the basis of structural motifs that were present in other natural product activators, we hypothesized that the halomadurones could be activators and would contribute further knowledge surrounding SAR. Therefore, halomadurones A–D (**1**–**4**) and 2-methyl-6-((*E*)-3-methyl-1,3-hexadiene)-γ-pyrone (**5**) were evaluated for Nrf2-ARE activation (Figure 2). *Tert*-butylhydroquinone (*t*BHQ) was used as a positive control. Halomadurones C (**3**) and D (**4**) demonstrated potent Nrf2-ARE activation in the hPAP assay, but toxicity was apparent at higher concentrations (Figure 2). Nrf2 activation did not increase in a dose-dependent manner with increasing concentrations of halomadurones C (**3**) and D (**4**) due to cytotoxicity. For example, treatment with concentrations of 7.5 and 15 µM resulted in greater Nrf2 activation than treatment with 30 µM. This decrease in activation at higher concentrations of halomadurones C (**3**) and D (**4**) correlated with an increase in cytotoxicity indicative of a narrow therapeutic window. More specifically, halomadurone D (**4**) had a dramatic decrease in hPAP activity at 30 µM in comparison to halomadurone C (**3**); yet, halomadurone D (**4**) had greater hPAP activity than halomadurone C (**3**) at 15 and 7.5 µM. Halomadurones A (**1**) and B (**2**) and 2-methyl-6-((*E*)-3-methyl-1,3-hexadiene)-γ-pyrone (**5**) demonstrated less than a ten-fold increase in Nrf2-ARE activation, considerably less than *t*BHQ. The four halogenated pyrones (**1**–**4**) demonstrated greater Nrf2 activation than the non-halogenated pyrone (**5**), suggesting the importance of the halogen atoms in their activity. Among the halogenated pyrones, brominated halomadurones C (**3**) and D (**4**) demonstrated a considerable increase in activation compared to chlorinated halomadurones A (**1**) and B (**2**), potentially due to the increased electrophilicity from the bromine atoms. However, at higher concentrations, electrophiles sometimes react with thiol groups, which can lead to toxicity [30]. Neuroprotection assays were conducted for halomadurone C (**3**) and D (**4**) at 3 µM, but neither compound demonstrated neuroprotection.

## 3. Experimental Section

### 3.1. General Experimental Procedures

Optical rotations were measured on a Perkin–Elmer 241 Polarimeter. UV spectra were recorded on an Aminco/OLIS UV-Vis spectrophotometer. IR spectra were measured with a Bruker Equinox 55/S FT–IR spectrophotometer. NMR spectra were obtained in CDCl_3_ with a Bruker Avance 600 MHz spectrometer equipped with a 1.7 mm ^1^H{^13^C/^15^N} cryoprobe, a Bruker Avance 500 MHz spectrometer equipped with a ^13^C/^15^N{^1^H} cryoprobe, and a Varian Unity-Inova 500 MHz spectrometer. HRMS data were acquired with a Bruker MaXis 4G QTOF mass spectrometer. RP HPLC was performed using a Shimadzu Prominence HPLC system and a Phenomenex Onyx Monolithic C18 column (100 × 4.6 mm).

### 3.2. Biological Material

Ascidian specimens were collected on 10 August 2011, in the Florida Keys (24°39.591′, 81°25.217′). A voucher specimen (FLK10-5-6) for *Ecteinascidia turbinata* (Herdman, 1880) is housed at the University of Wisconsin-Madison. For cultivation, a sample of ascidian (1 cm^3^) was rinsed with sterile seawater, macerated using a sterile pestle in a micro-centrifuge tube, and dilutions were made in sterile seawater, with vortexing between steps to separate bacteria from heavier tissues. Dilutions were separately plated on three media: ISP2, R2A, and M4. Each medium was supplemented with 50 µg/mL cycloheximide and 25 µg/mL nalidixic acid. Plates were incubated at 28 °C for at least 28 days, and strain WMMB499 was purified from an ISP2 isolation plate.

### 3.3. Sequencing

16S rDNA sequencing was conducted as previously described [31]. WMMB499 was identified as an *Actinomadura* sp. and demonstrated 99% sequence similarity to *Actinomadura* sp. 13679C (accession number EU741239). The 16S sequence for WMMB499 was deposited in GenBank (accession number JX101467). 

### 3.4. Fermentation, Extraction, and Isolation

Two 10 mL seed cultures (25 × 150 mm tubes) in medium ASW-A (20 g soluble starch, 10 g glucose, 5 g peptone, 5 g yeast extract, 5 g CaCO_3_ per liter of artificial seawater) were inoculated with strain WMMB499 and shaken (200 RPM, 28 °C) for seven days. Two hundred fifty mL baffled flasks (12 × 50 mL) containing ASW-A were inoculated with 1 mL seed culture and were incubated (200 RPM, 28 °C) for seven days. Two-liter flasks (6 × 500 mL) containing medium ASW-A with Diaion HP20 (4% by weight) were inoculated with 25 mL from the 50 mL culture and shaken (200 RPM, 28 °C) for seven days. Filtered HP20 and cells were washed with H_2_O and extracted with acetone. The acetone extract (3.5 g) was subjected to liquid-liquid partitioning using 30% aqueous MeOH and CHCl_3_ (1:1). The CHCl_3_-soluble partition (2.5 g) was fractionated by Sephadex LH20 column chromatography (column size CHCl_3_:MeOH, 1:1). Fractions containing **1** and **2** were subjected to RP HPLC (55%/45% to 75%/25% MeOH/H_2_O, 15 min, 3 mL/min) using a Phenomenex Onyx Monolithic C18 column (100 × 4.6 mm), yielding **2** (3.0 mg, *t*_R_ 7.1 min) and **1** (3.2 mg, *t*_R_ 11.0 min). For production of **3** and **4**, the amount of KBr in medium ASW-A was increased from 0.1 g/L to 10 g/L, and no NaCl was included. After fermentation of 500 mL ASW-A (with KBr), the same isolation procedure was used. One additional purification step by RP HPLC (70%/30% to 76%/24% ACN/H_2_O, 11 min, 4 mL/min) using a Phenomenex Luna C18 column (250 × 10 mm, 5 µm) yielded **3** (1.3 mg, *t*_R_ 8.8 min) and **4** (1.2 mg, *t*_R_ 9.1 min).

Halomadurone A (**1**). White solid; UV (MeOH) λ_max_ (log ε) 204 (3.96), 223 (3.99), 255 (3.90), 315 (3.92) nm; IR (ATR) ν_max_ 1644, 1635, 1394, 1215 cm^−1^; ^1^H and ^13^C NMR (See Table 1); HRMS [M + H]^+^ *m/z* 307.0044 (calcd. for C_13_H_14_Cl_3_O_2_, 307.0054).

Halomadurone B (**2**). White solid; UV (MeOH) λ_max_ (log ε) 204 (4.04), 222 (3.94), 253 (3.79), 315 (3.83) nm; IR (ATR) ν_max_ 1641, 1632, 1602, 1369, 932 cm^−1^; ^1^H and ^13^C NMR (See Table 1); HRMS [M + H]^+^ *m/z* 273.0453 (calcd. for C_13_H_15_Cl_2_O_2_, 273.0444).

Halomadurone C (**3**). White solid; 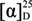 ±0 UV (MeOH) λ_max_ (log ε) 206 (4.02), 253 (3.40), 324 (3.50) nm; IR (ATR) ν_max_ 1650, 1601, 1396, 1215, 934 cm^−1^; ^1^H and ^13^C NMR (See Table 1); HRMS [M + H]^+^ *m/z* 316.9937 (calcd. for C_13_H_15_BrClO_2_, 316.9938).

Halomadurone D (**4**). White solid; (*c* 0.12, MeOH); UV (MeOH) λ_max_ (log ε) 206 (4.30), 255 (3.42), 324 (3.42) nm; IR (ATR) ν_max_ 1651, 1601, 1396, 1215, 934 cm^−1^; ^1^H and ^13^C NMR (See Table 1); HRMS [M + H]^+^ *m/z* 360.9433 (calcd. for C_13_H_15_Br_2_O_2_, 360.9433).

2-Methyl-6-((*E*)-3-methyl-1,3-hexadiene)-γ-pyrone (5). To a solution of 2-6-dimethyl-γ-pyrone (1.24 g) in 15 mL ethanol was added 2-methyl-2-pentenal (2.28 mL) and sodium ethoxide (680 mg) in 7.5 mL ethanol. The reaction mixture was stirred for 5 h at rt. After 5 h, dilute HCl was added to the mixture and dried under vacuum. The reaction mixture was subjected to RP HPLC (40%/60% to 100%/0% MeOH/H_2_O containing 0.1% acetic acid, 20 min) using a Phenomenex Gemini C18 column (100 × 30 mm, 5 µm). Additional purification of the fraction containing 5 was conducted by Normal Phase HPLC (60%/40% to 100%/0% ethyl acetate/hexanes, 30 min) using a Phenomenex Luna Silica column (250 × 10 mm, 5 µm), yielding 5 (4.0 mg, 2.0% yield, *t*_R_ 25.5 min) as a white solid; UV (MeOH) λ_max_ (log ε) 208 (3.99), 227 (4.00), 314 (4.17) nm; IR (ATR) ν_max_ 1655, 1597, 1398, 1217, 925 cm^−1^; ^1^H and ^13^C NMR (See Supporting Information); HRMS [M + H]^+^ *m/z* 205.1230 (calcd. for C_13_H_17_O_2_, 205.1223). 

### 3.5. Bioasssay hPAP and MTS

Primary cortical neuronal cultures were derived from ARE-hPAP reporter mice as previously described [10,11]. Compounds were dissolved in 100% DMSO and administered to cells for 48 h. *Tert*-butylhydroquinone (*t*BHQ) was used as a control. After 48 h, Nrf2 activation was determined by measuring for hPAP activity as previously described [32]. Using one-second integration luminescence was measured on a Berthold Orion microplate luminometer (Berthold Technologies GmbH & Co., Bad Wildbad, Germany). Baseline signals from hPAP negative control culture samples were subtracted from all values. Cell viability was assayed using the MTS assay following the manufacturer’s suggested protocol (Promega, Madison, Wisconsin, USA). All hPAP and MTS data are represented as mean ± SEM (*n* = 4). Results of hPAP assays are expressed as the fold increase in hPAP activity over basal levels. Statistical analysis was performed using one-way ANOVA followed by Newman–Keuls multiple comparison (GraphPad Prism, version 4). A *p* < 0.05 was considered statistically significant. 

## 4. Conclusions

We reported the isolation and structure elucidation of halomadurones A–D (**1**–**4**), novel halogenated electrophilic pyrones from an *Actinomadura* sp. and the synthesis of a non-halogenated analogue, 2-methyl-6-((*E*)-3-methyl-1,3-hexadiene)-γ-pyrone (**5**). Halomadurones C (**3**) and D (**4**) demonstrated potent Nrf2-ARE activation. Therefore, halomaudrones A–D (**1**–**4**) could play an important role in the discovery of new therapeutics, especially considering the ever-present need for therapeutics for neurodegenerative diseases. 

## Supplementary Files

Supplementary File 1 Supplementary Materials (PDF, 503 KB)
