# Supplementary material for: Activation of the Nuclear Factor E2-Related Factor 2 Pathway by Novel Natural Products Halomadurones A–D and a Synthetic Analogue
Source: Mar Drugs. 2013 Dec 16;11(12):5089–99. doi: 10.3390/md11125089 (PMC3877905; doi:10.3390/md11125089)

Supplementary Materials

Figure S1. ^{1}H NMR (600 MHz, $CDCl_3$) of **1**.

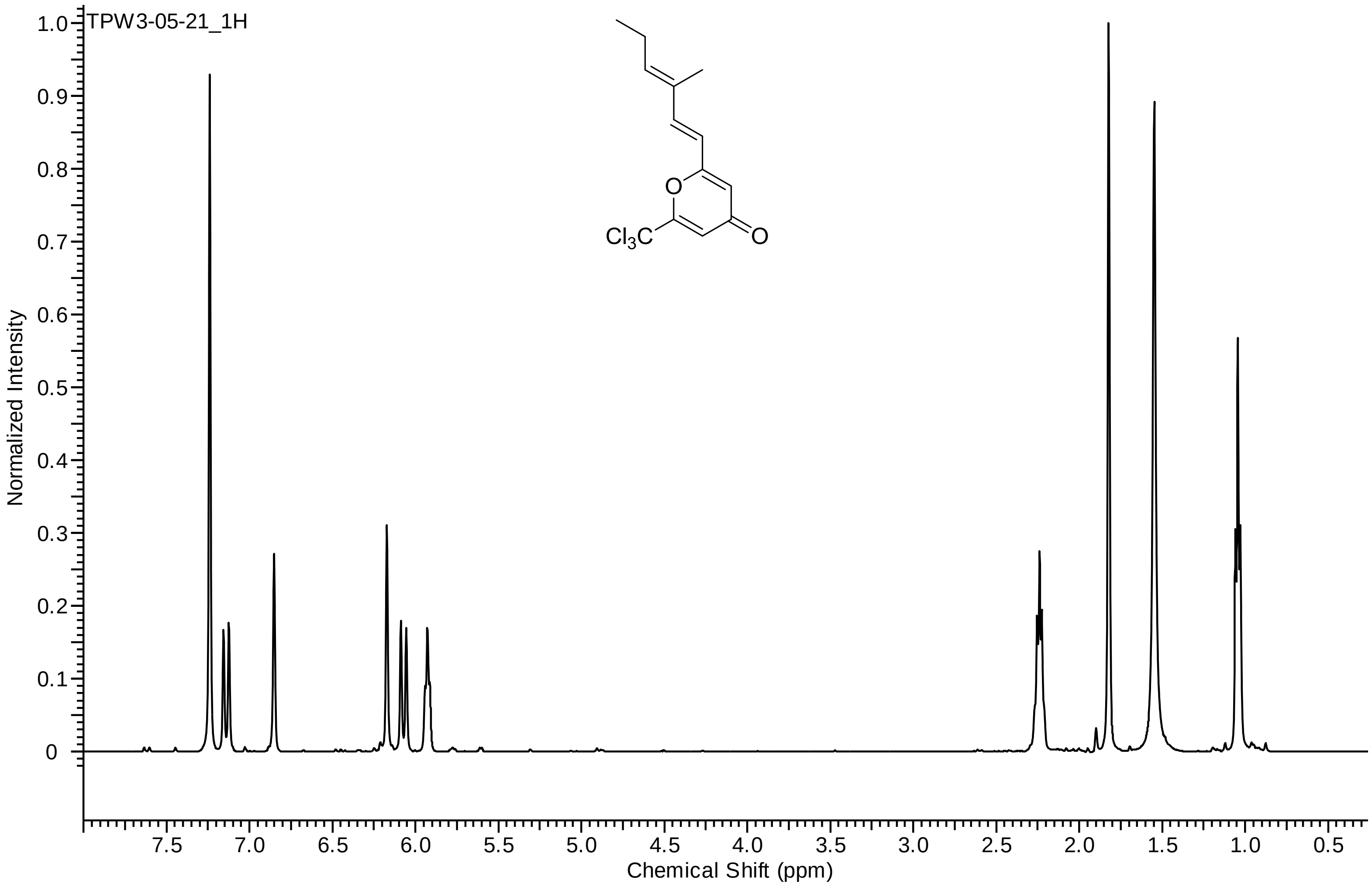

Figure S2. ^{13}C NMR (125 MHz, $CDCl_3$) of **1**.

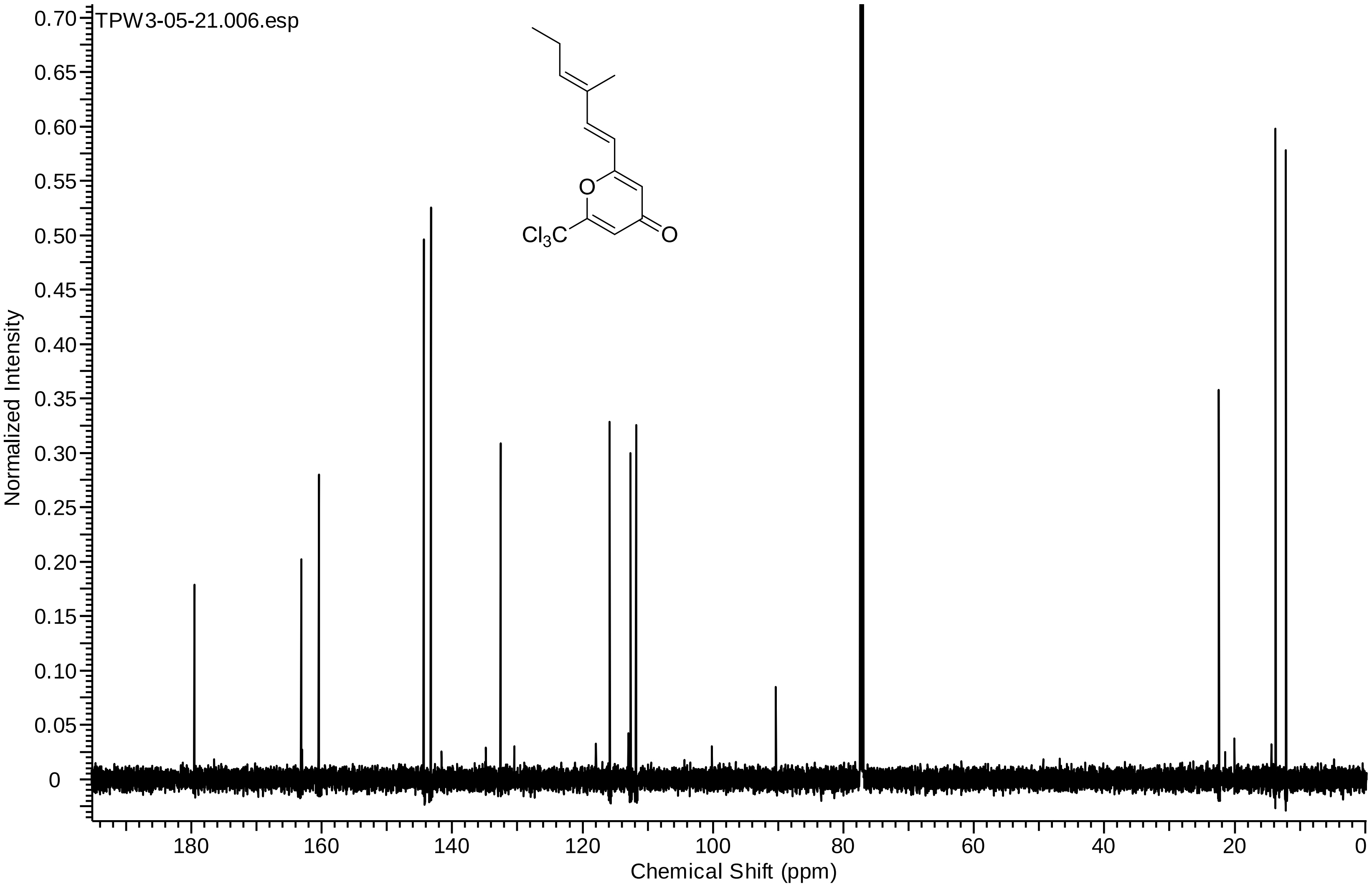

Figure S3. gCOSY (600 MHz, $CDCl_3$) of **1**.

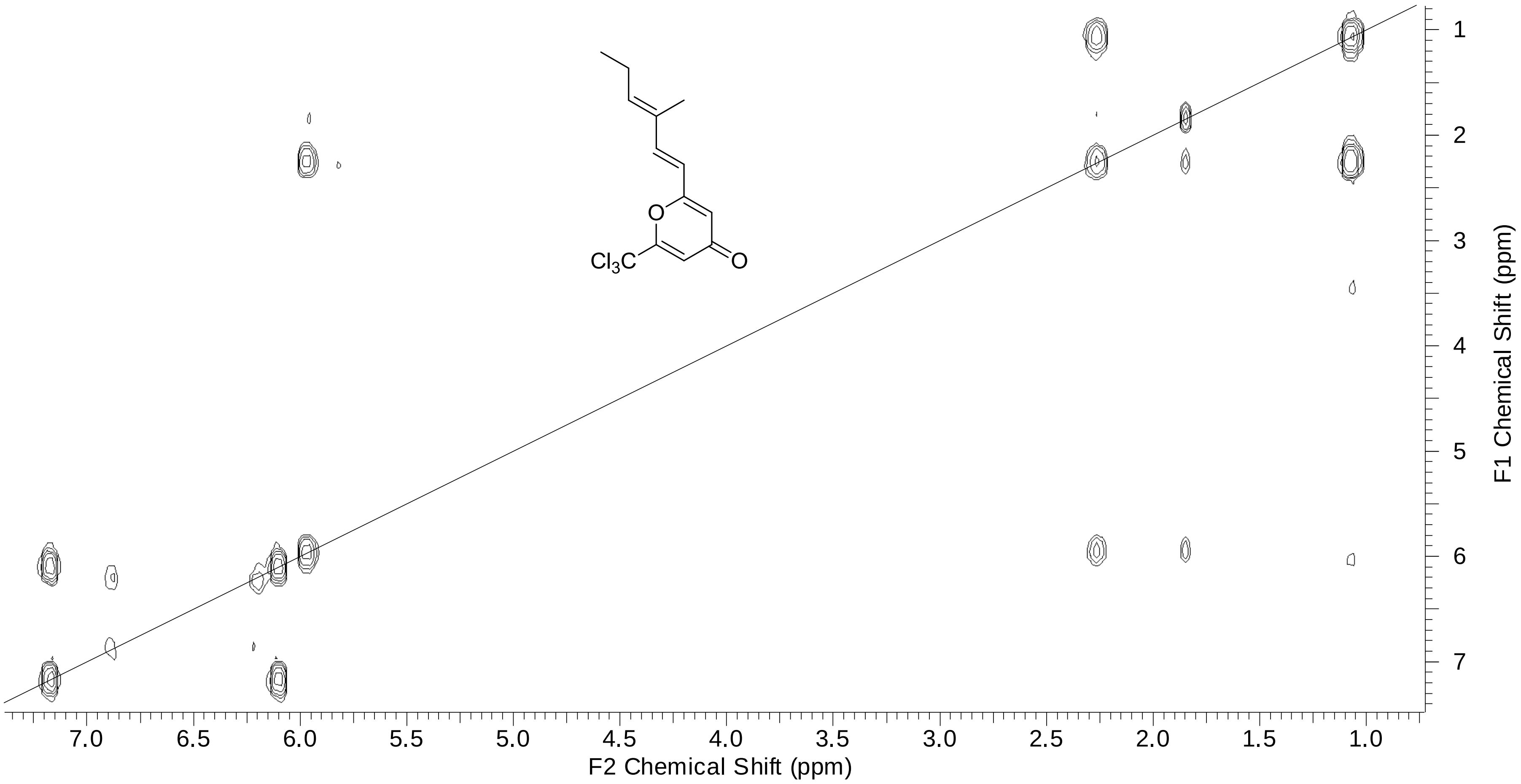

Figure S4. gHSQC (600 MHz, $CDCl_3$) of **1**.

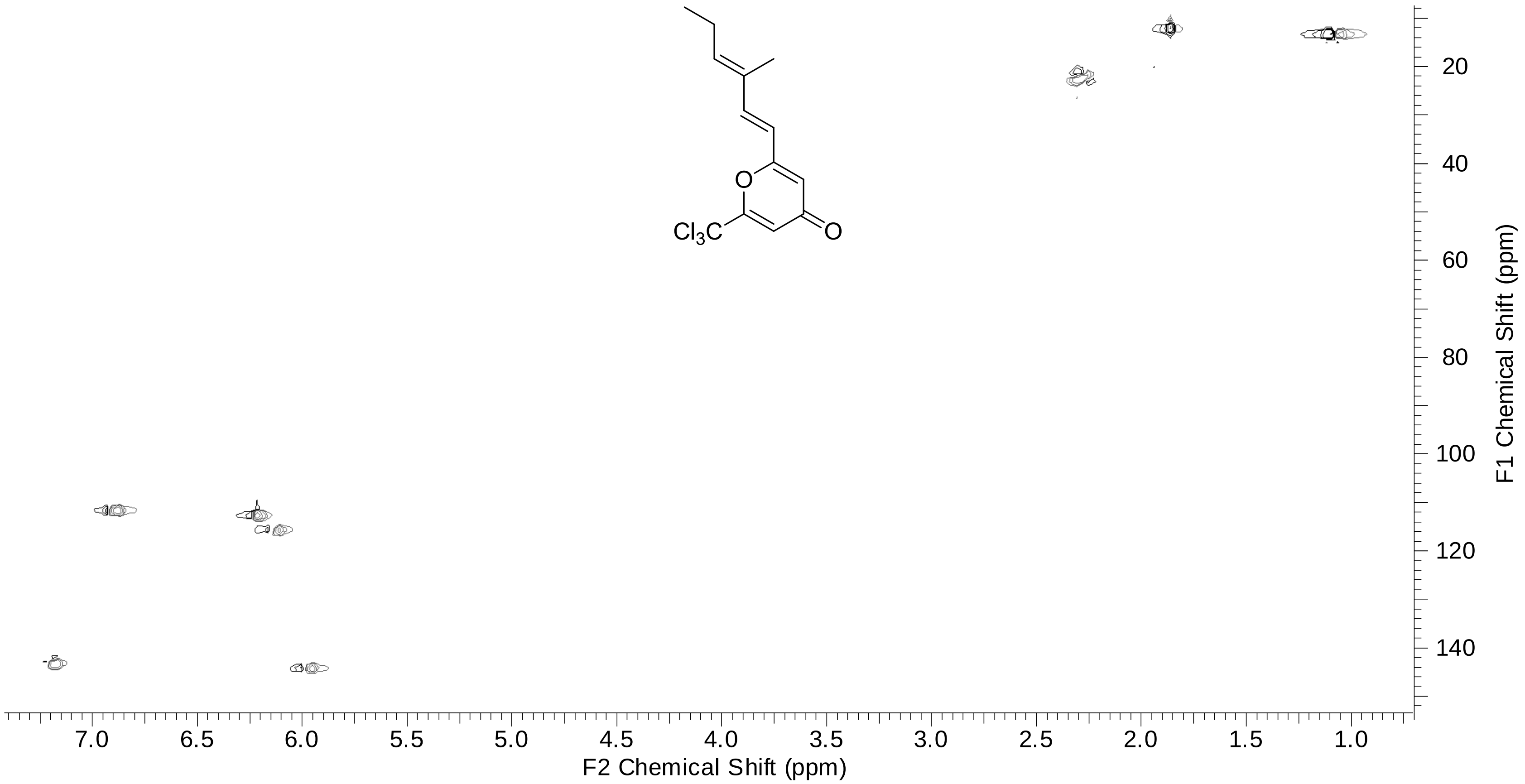

Figure S5. gHMBC (600 MHz, $CDCl_3$) of **1**.

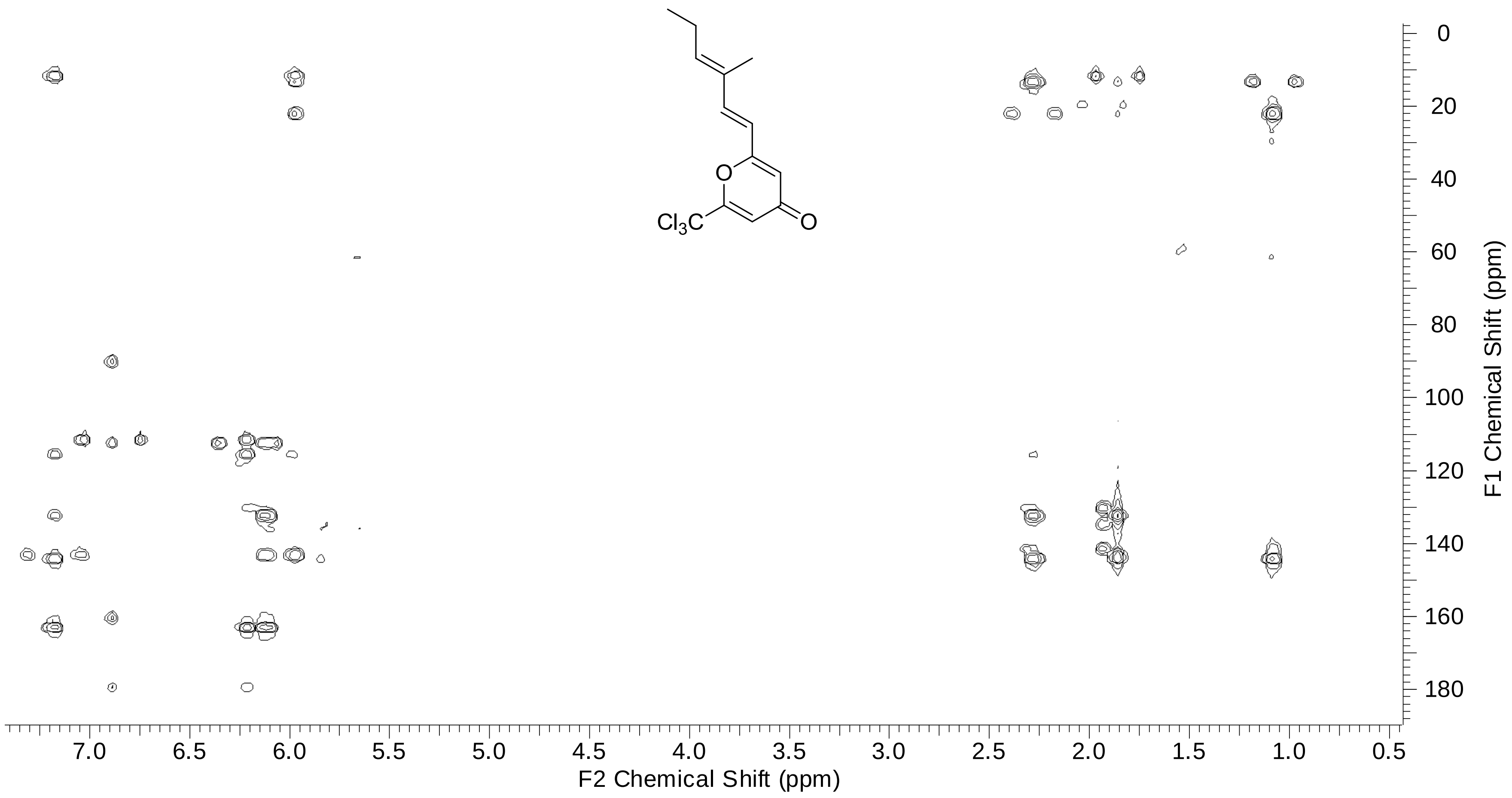

Figure S6. HRMS of **1**.

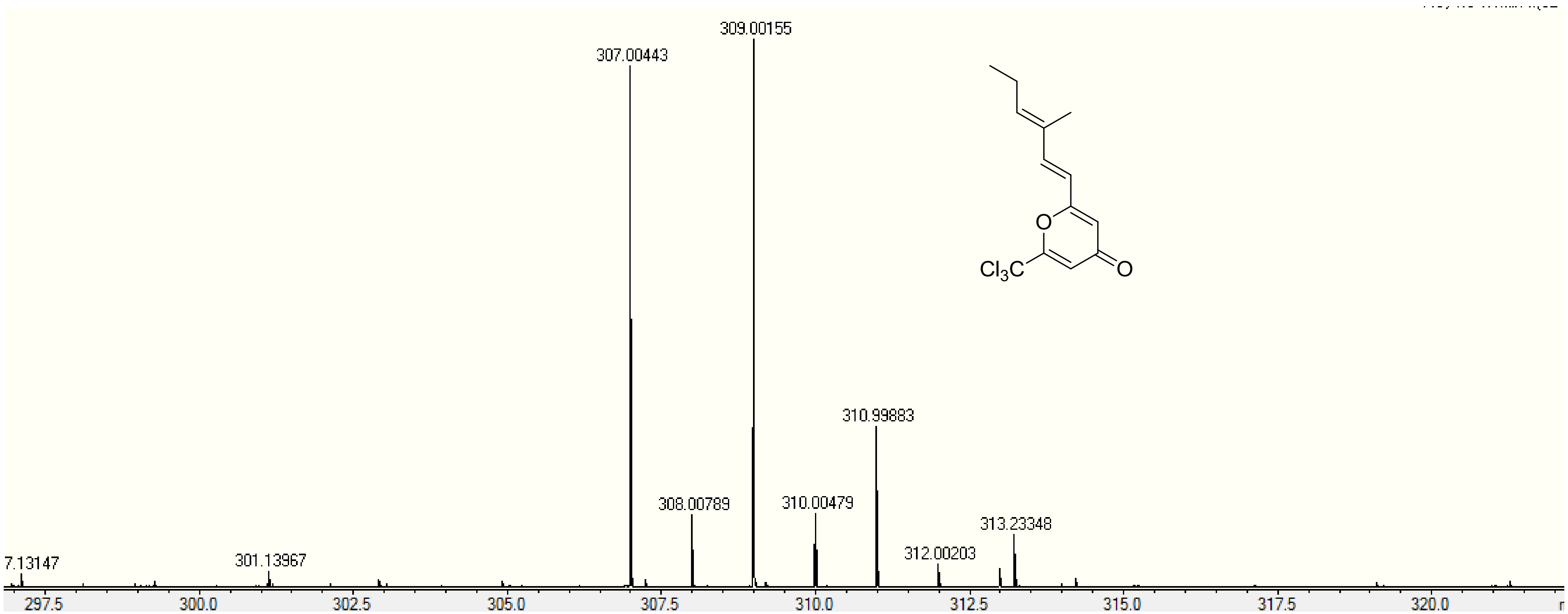

Figure S7. ^{1}H NMR (600 MHz, $CDCl_3$) of **2**.

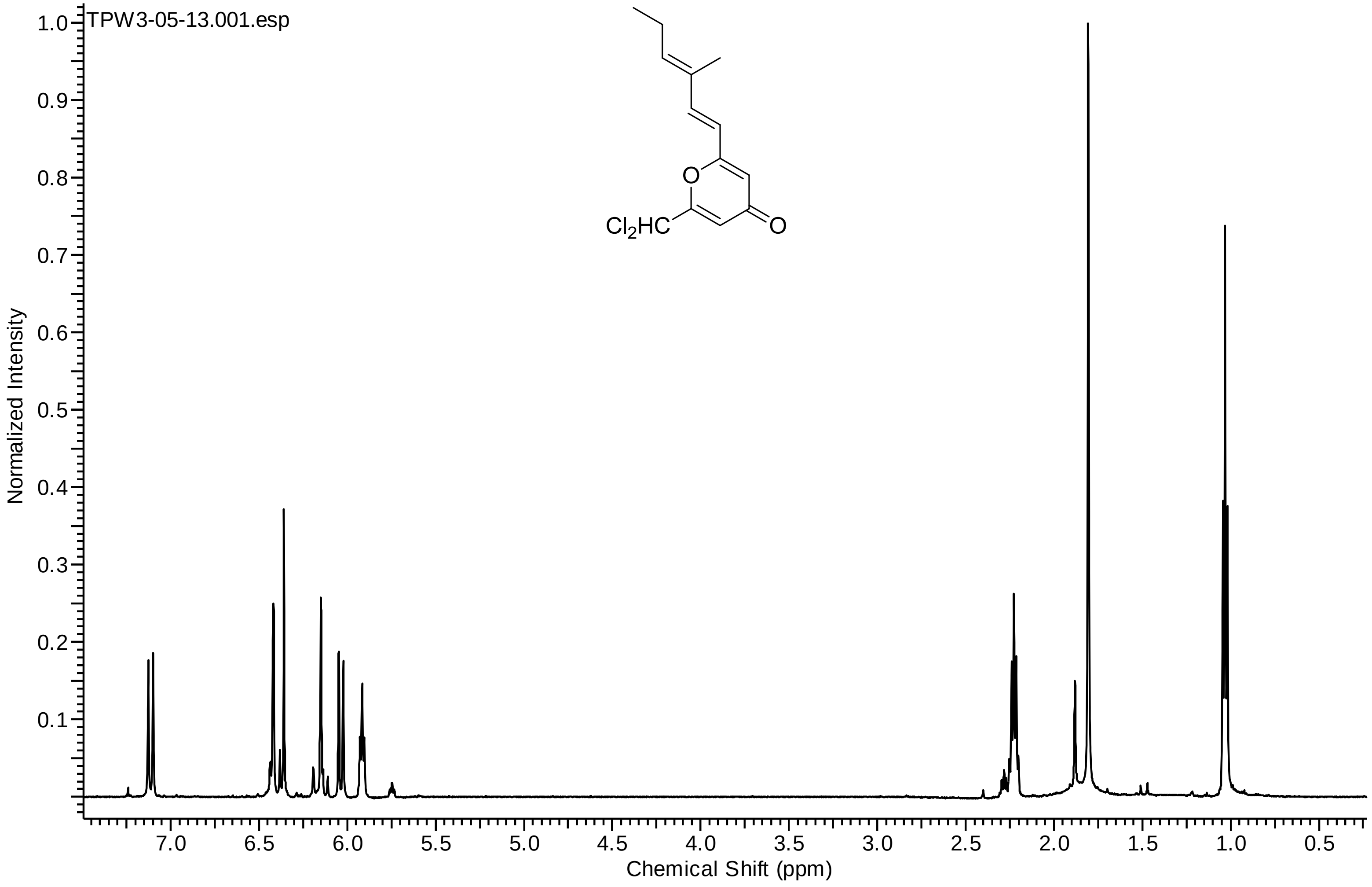

Figure S8. ^{13}C NMR (125 MHz, $CDCl_3$) of **2**.

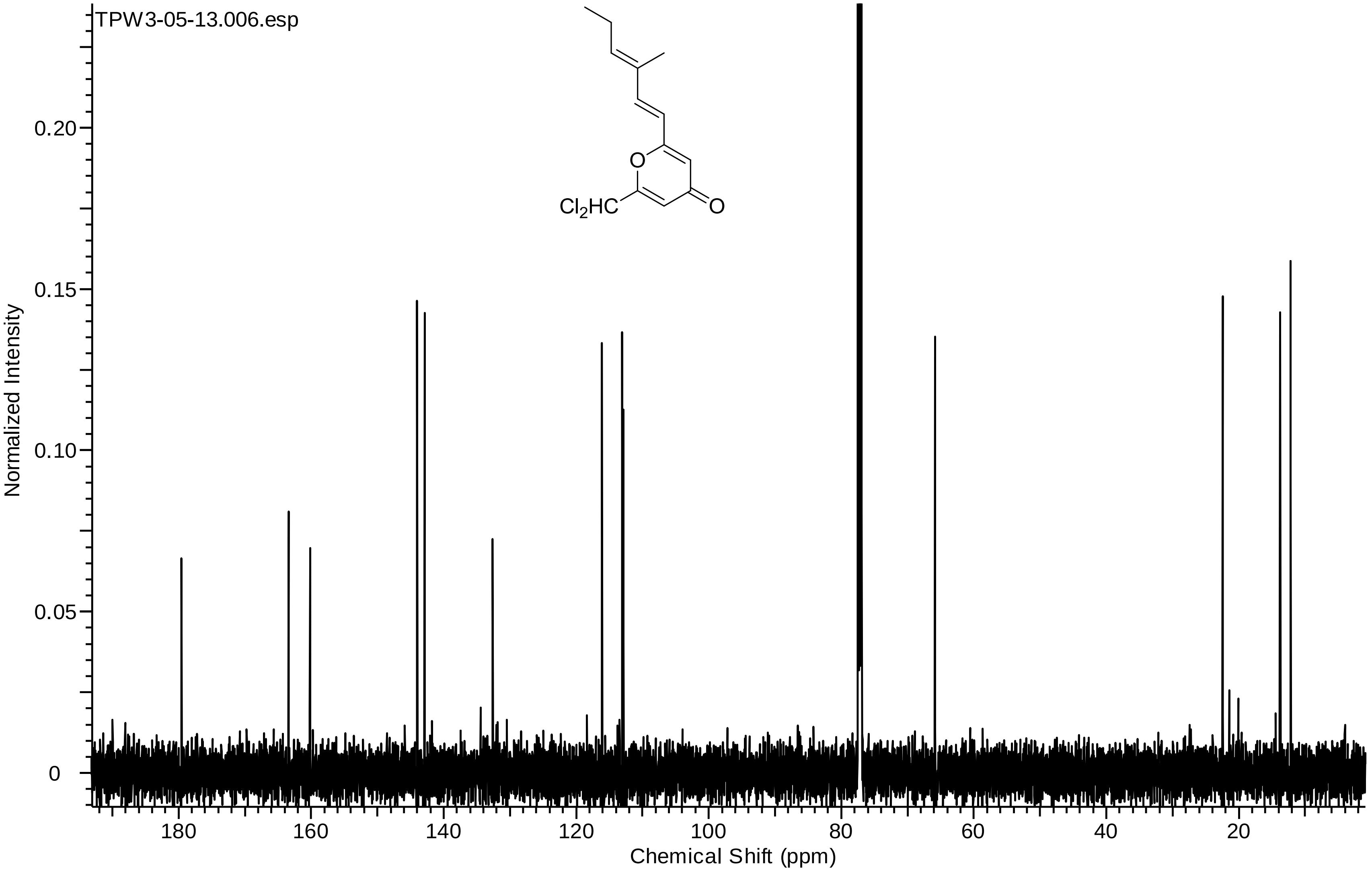

Figure S9. gCOSY (600 MHz, $CDCl_3$) of **2**.

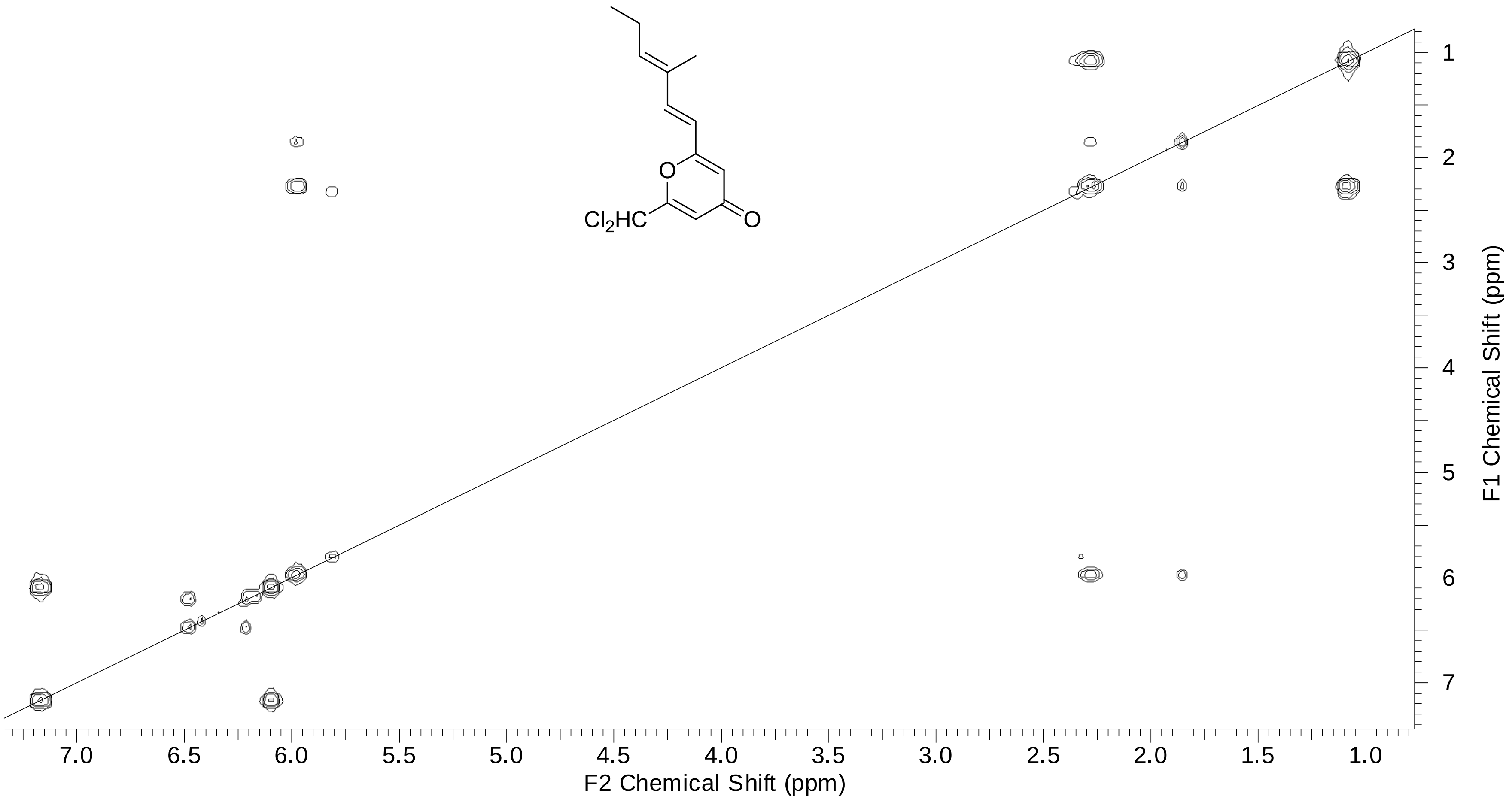

Figure S10. gHSQC (600 MHz, $CDCl_3$) of **2**.

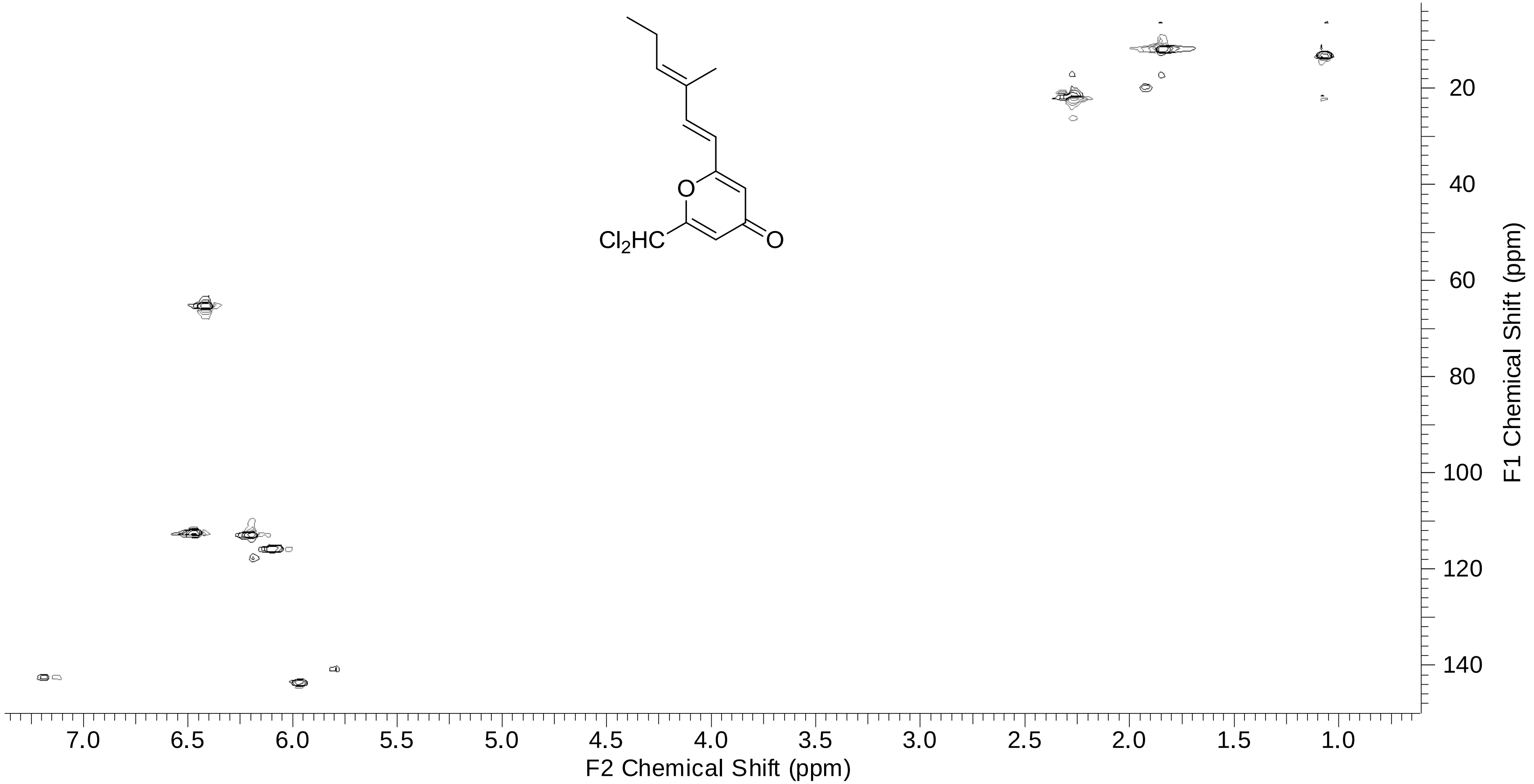

Figure S11. gHMBC (600 MHz, $CDCl_3$) of **2**.

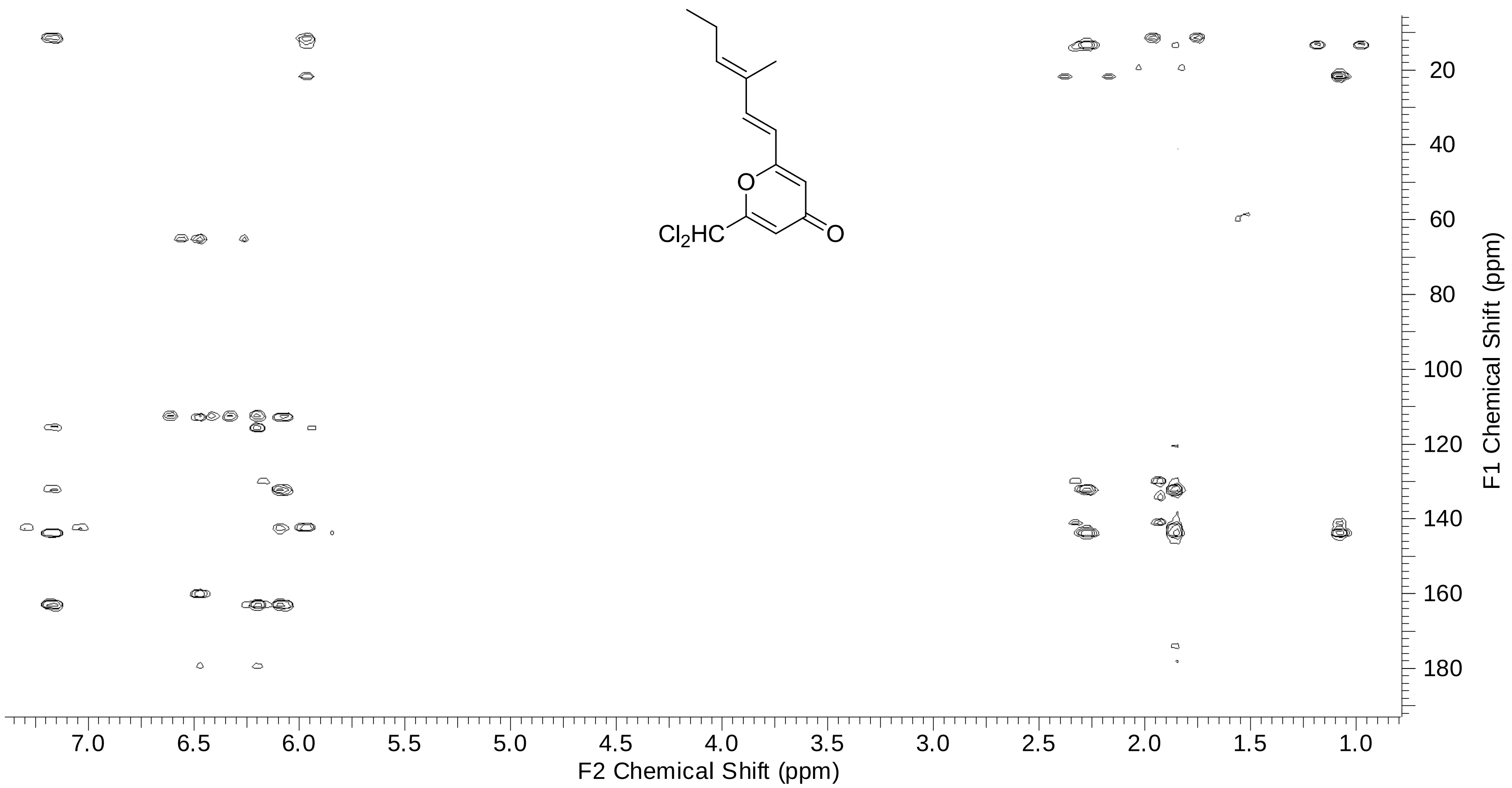

Figure S12. HRMS of **2**.

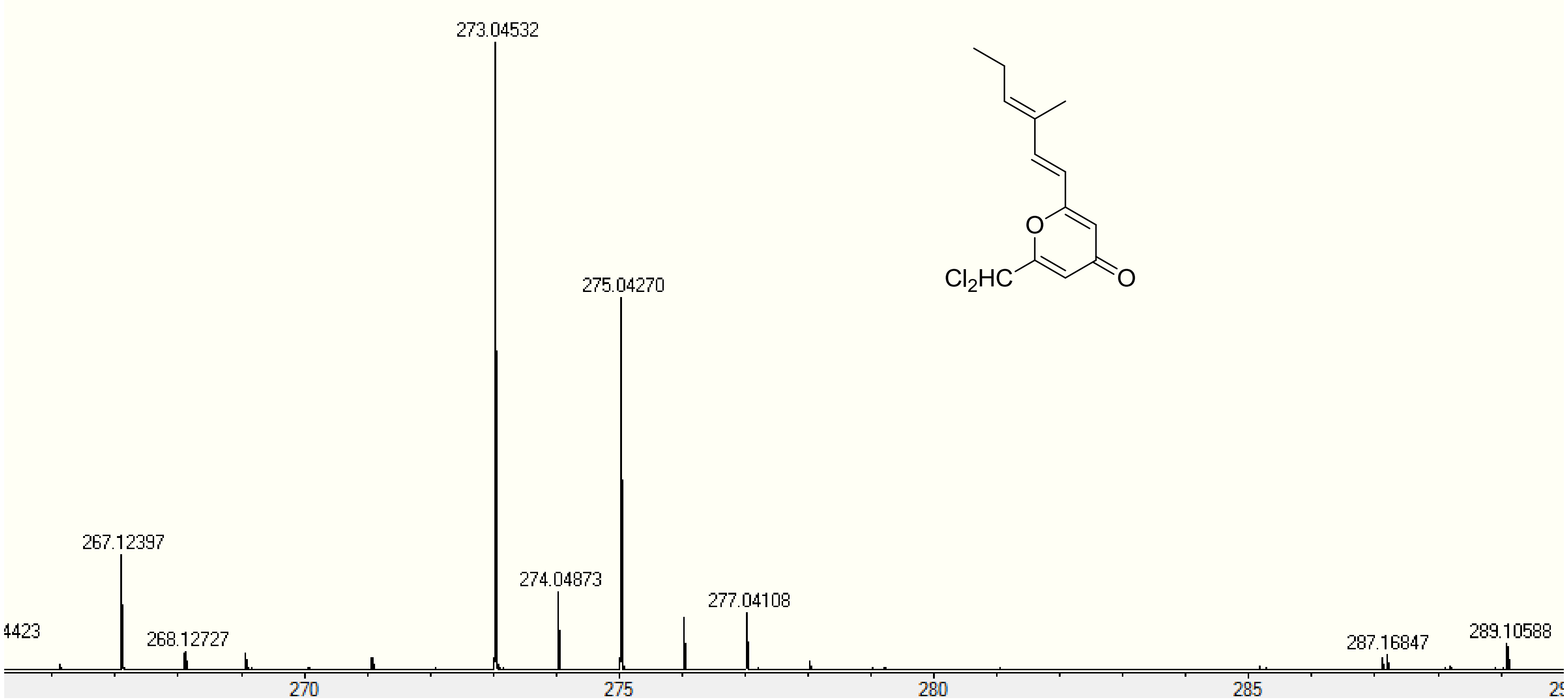

Figure S13. Key ROESY correlations.

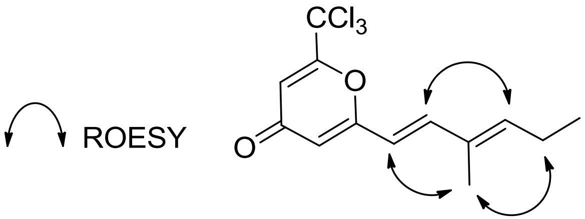

Figure S14. ^{1}H NMR (600 MHz, $CDCl_3$) of **3**.

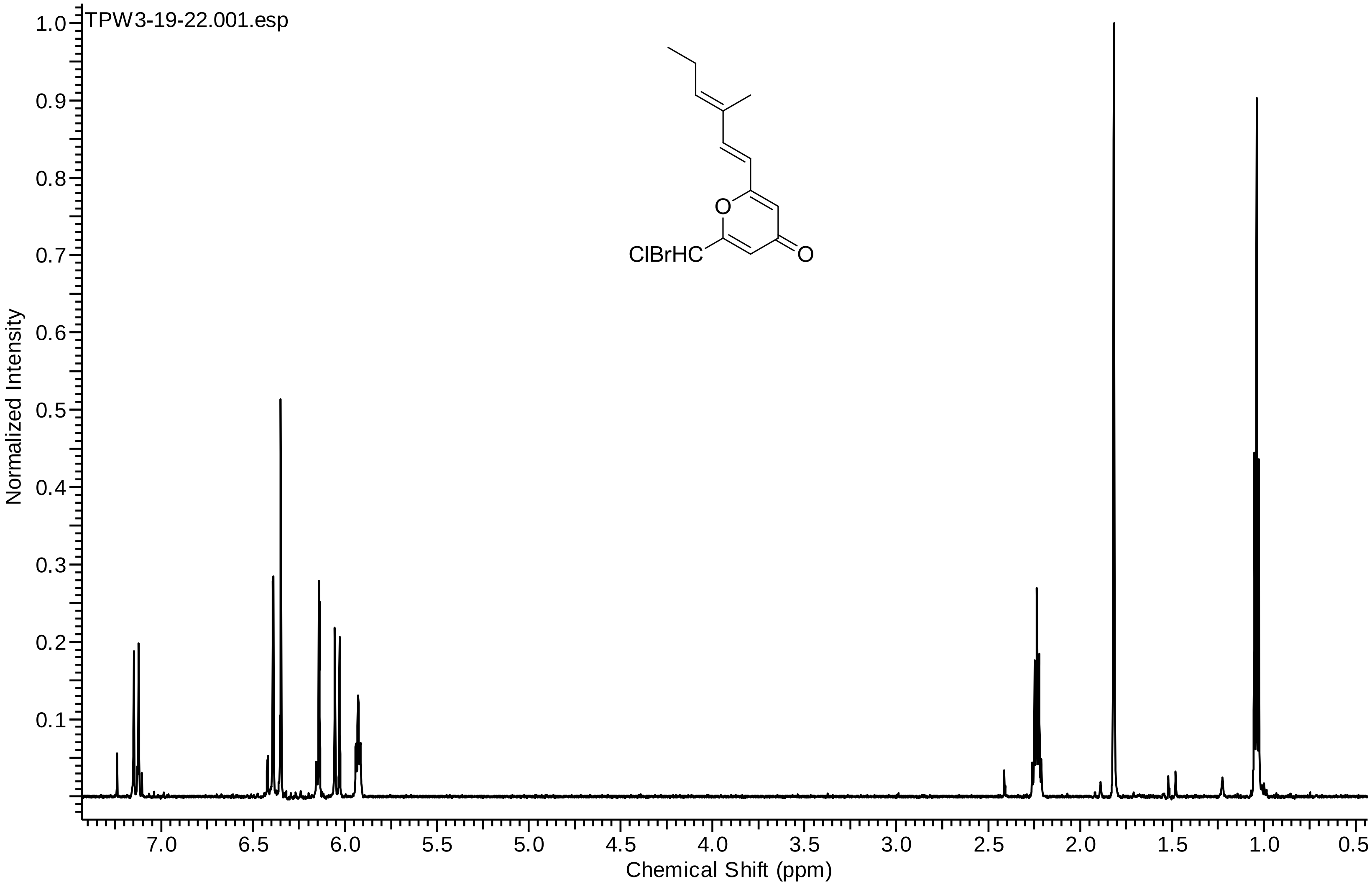

Figure S15. ^{13}C NMR (125 MHz, $CDCl_3$) of **3**.

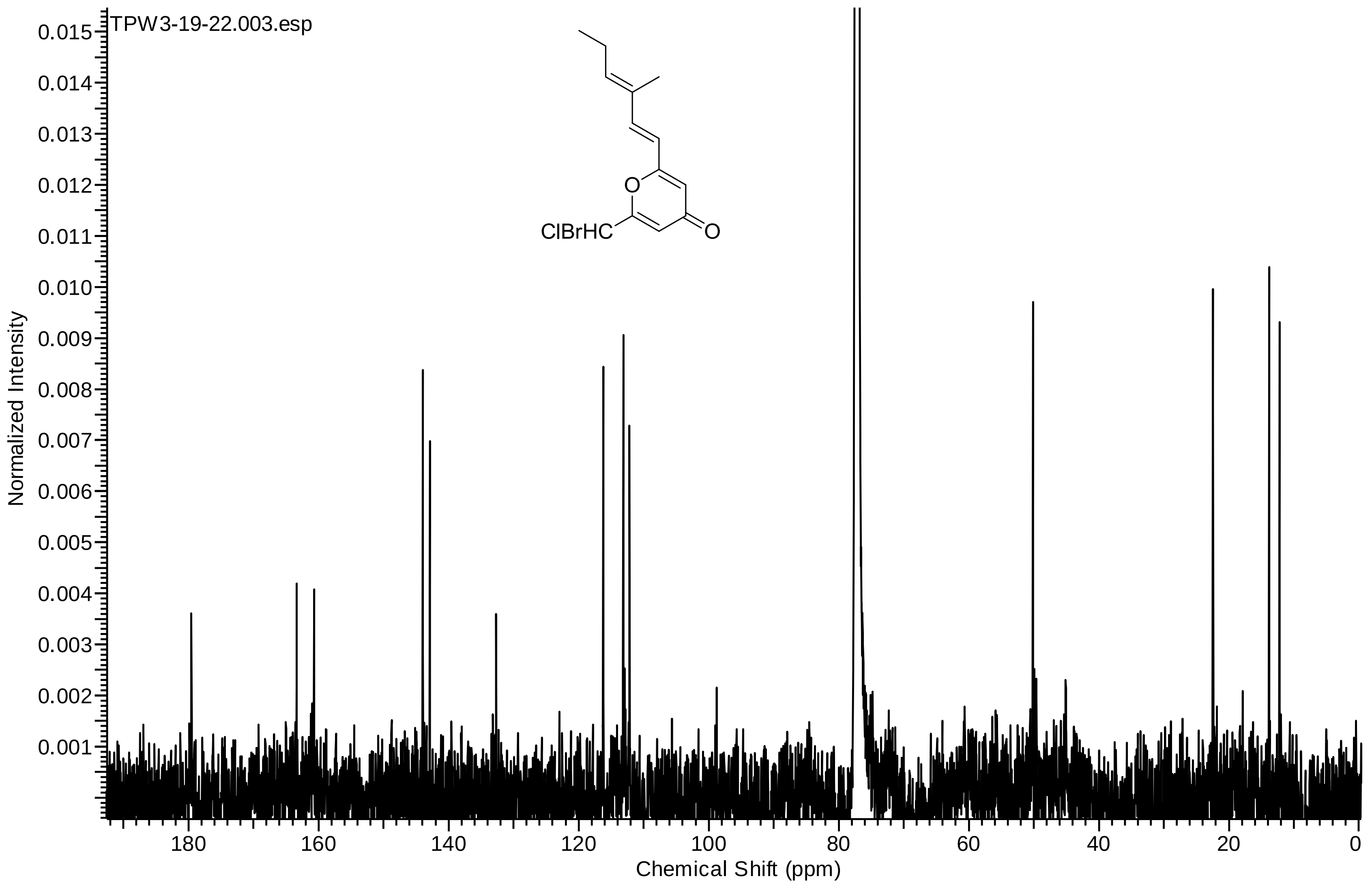

Figure S16. gCOSY (600 MHz, $CDCl_3$) of **3**

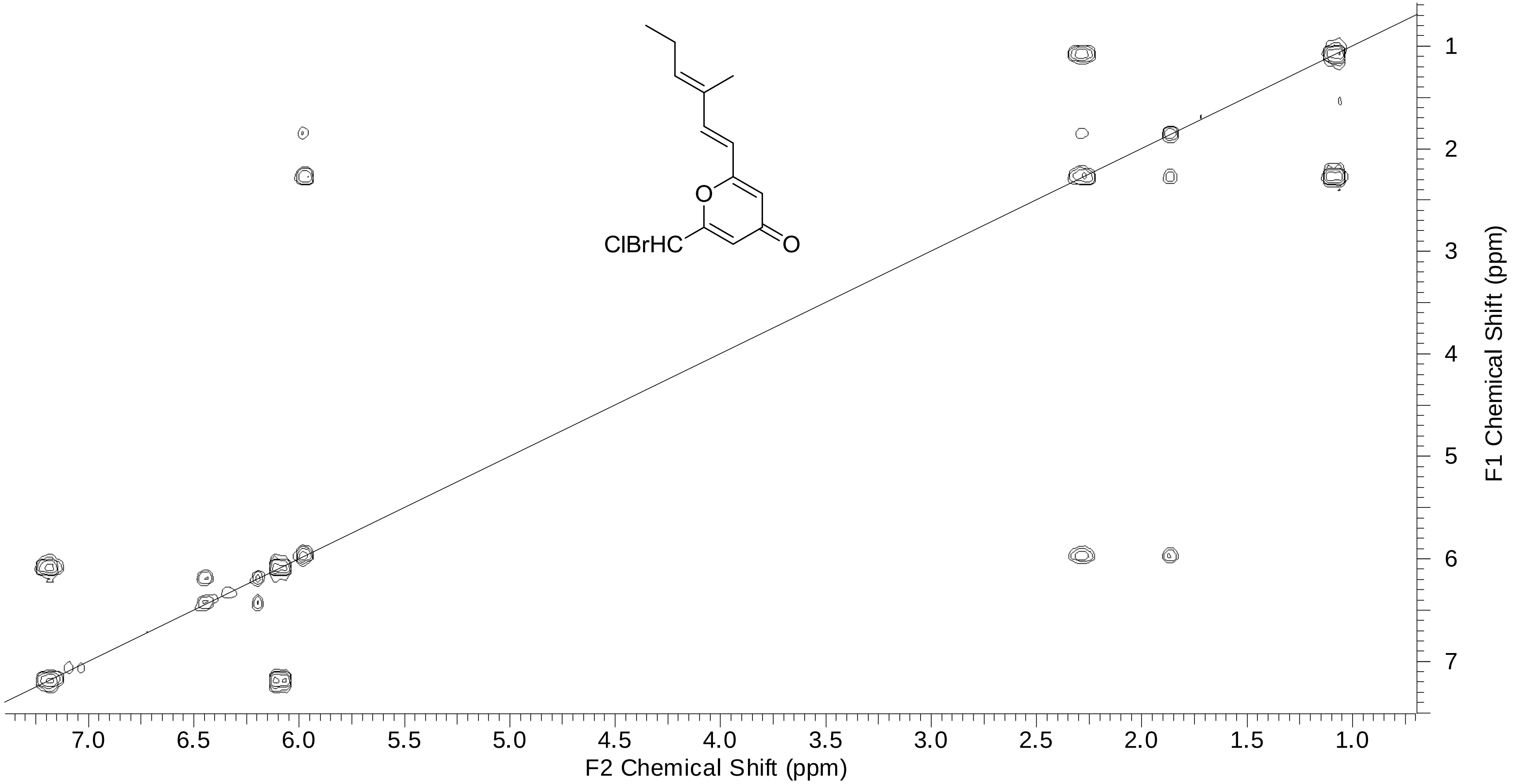

Figure S17. gHSQC (600 MHz, $CDCl_3$) of **3**.

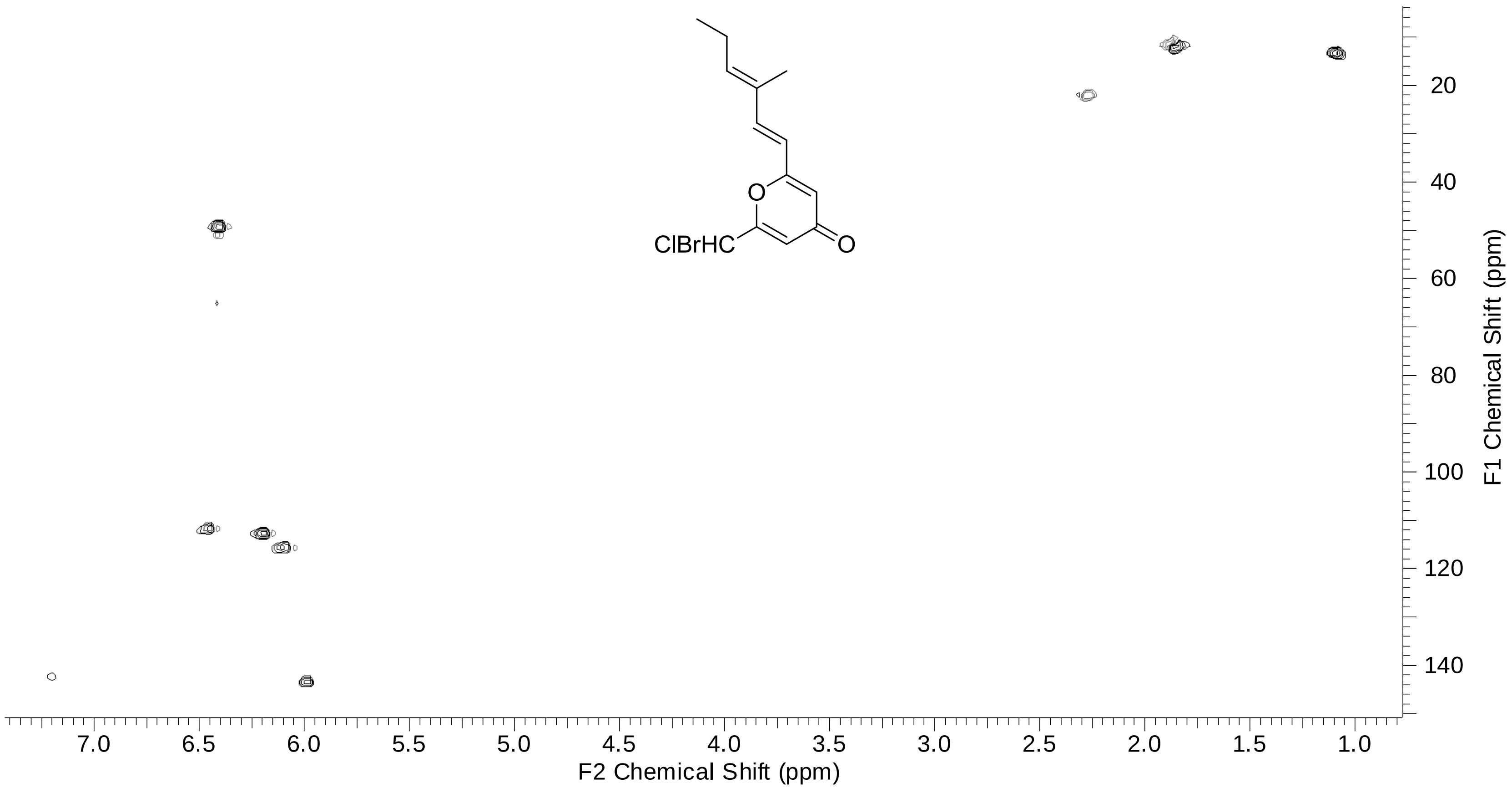

Figure S18. gHMBC (600 MHz, $CDCl_3$) of **3**.

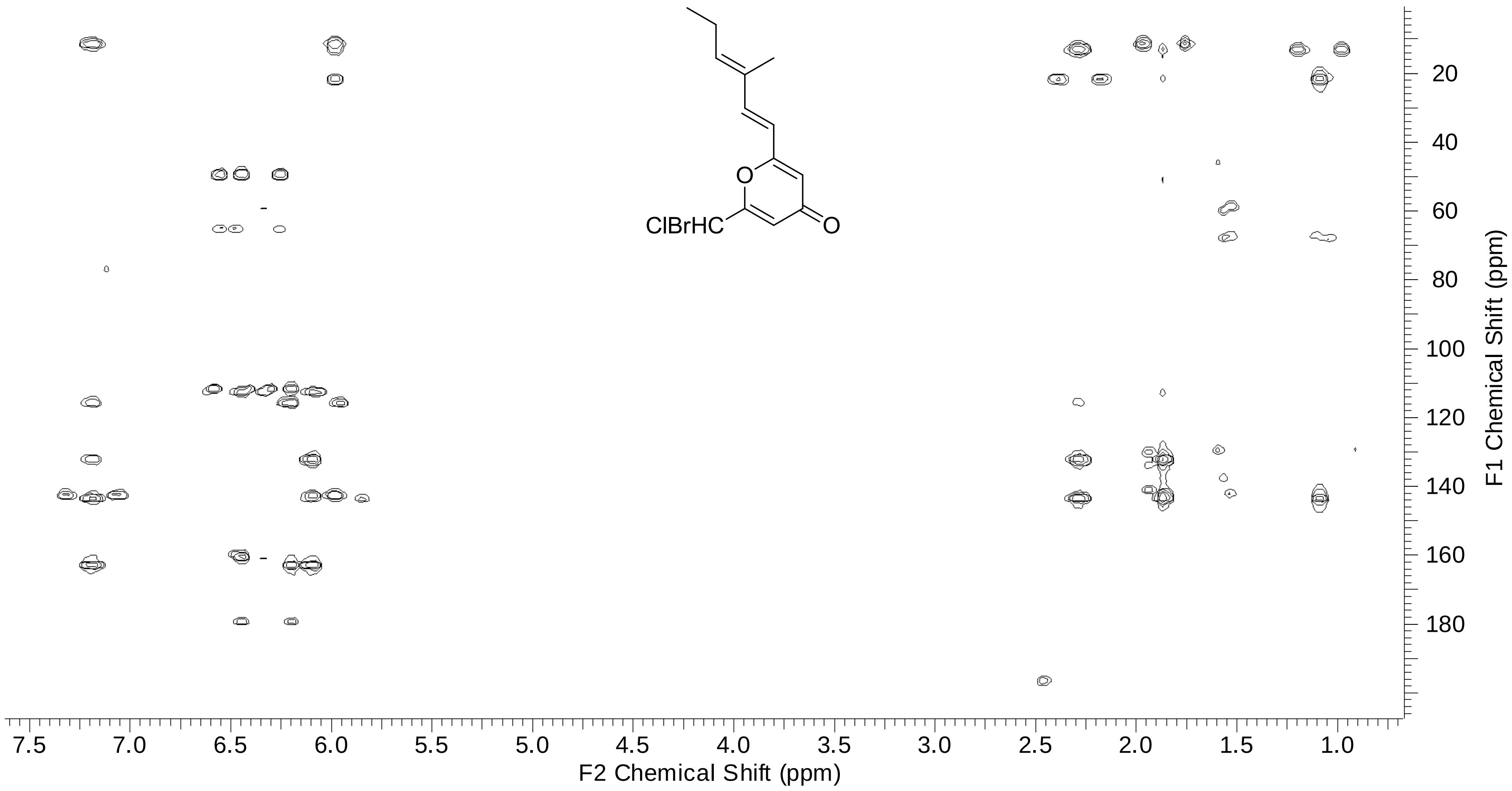

Figure S19. HRMS of **3**.

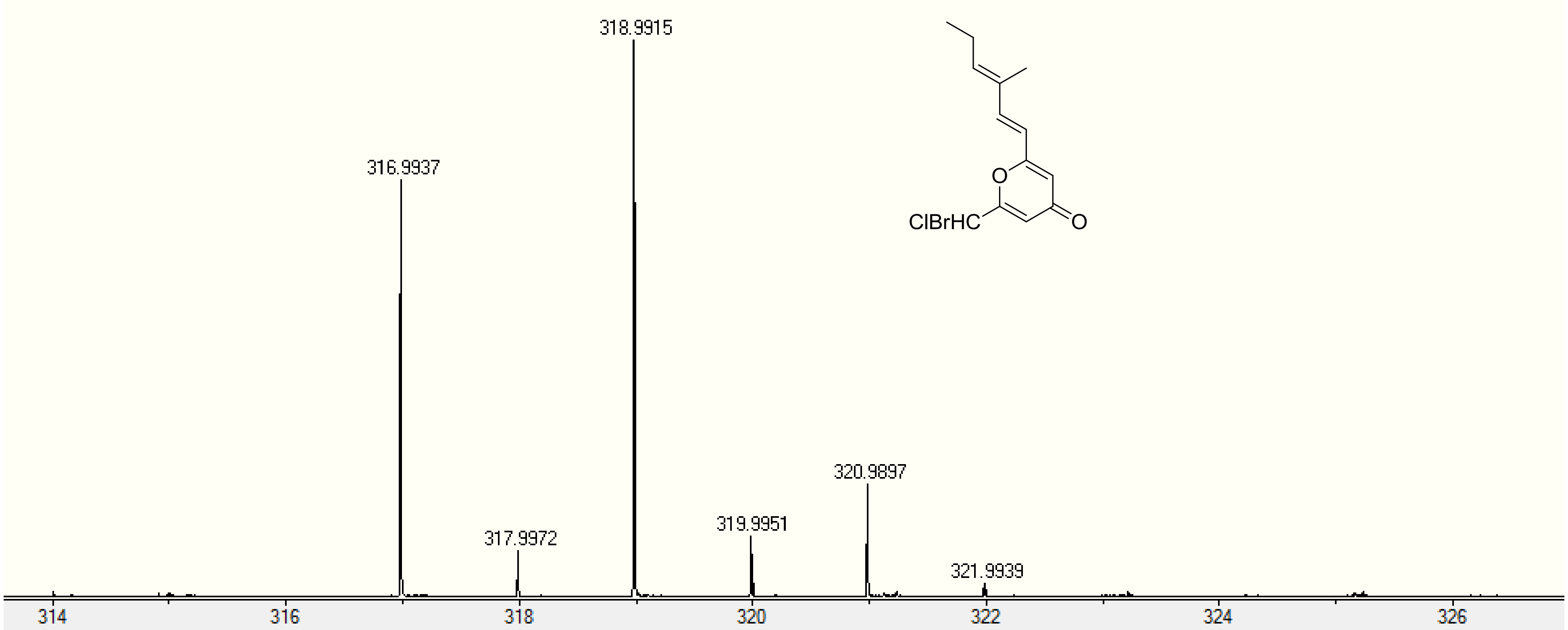

Figure S20. ^{1}H NMR (600 MHz, $CDCl_3$) of **4**.

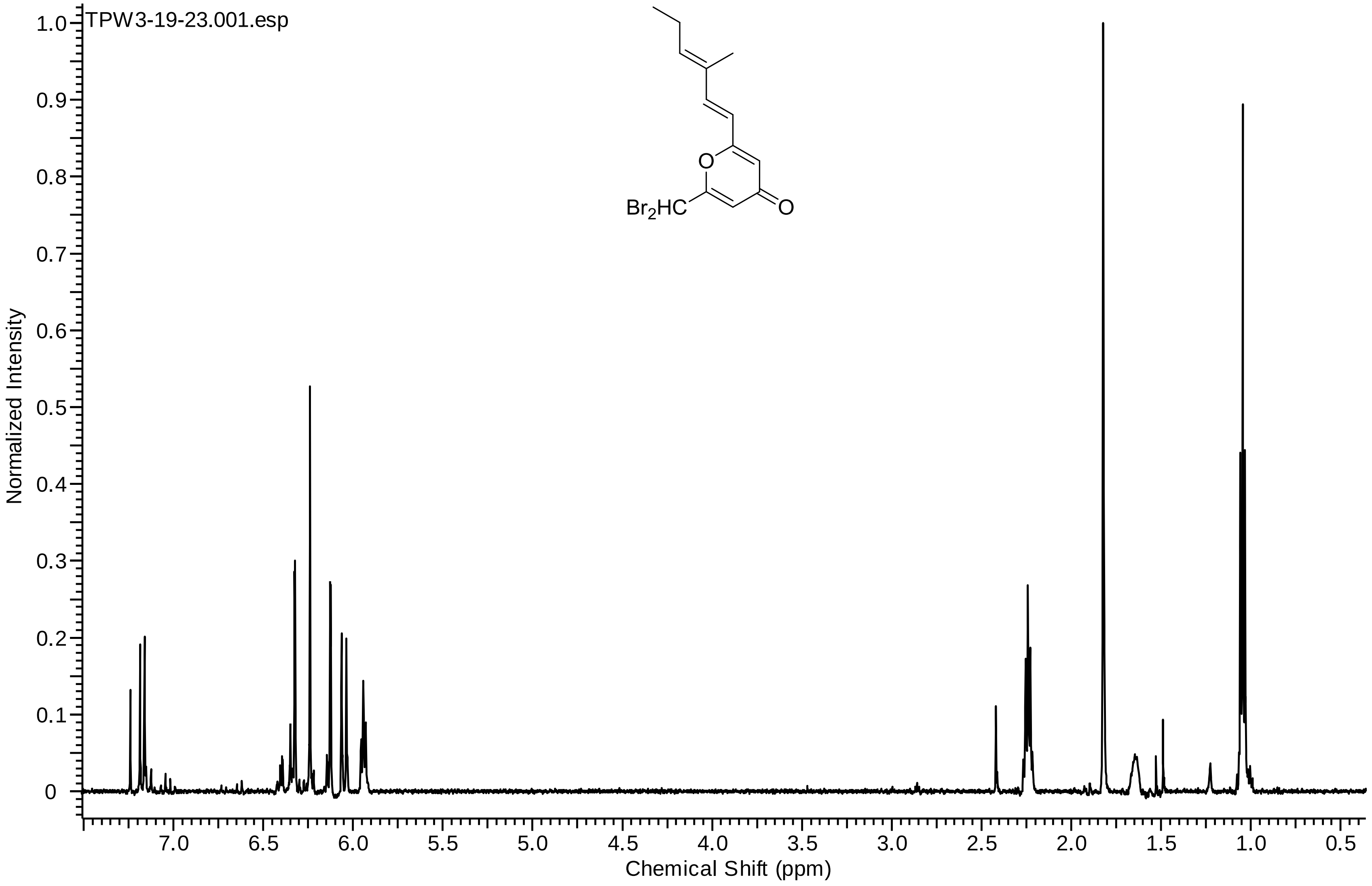

Figure S21. ^{13}C NMR (125 MHz, $CDCl_3$) of **4**.

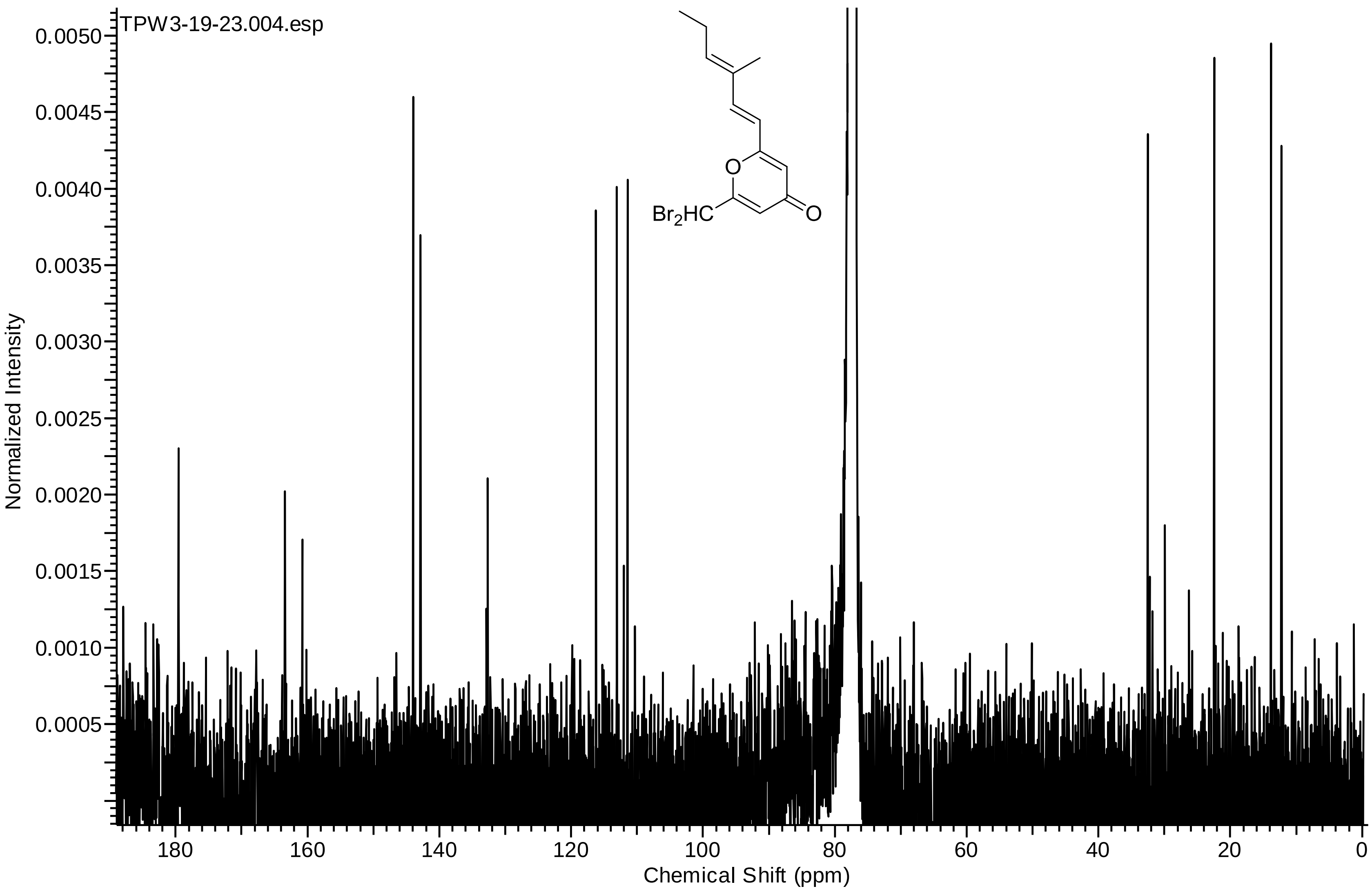

Figure S22. gCOSY (600 MHz, $CDCl_3$) of **4**.

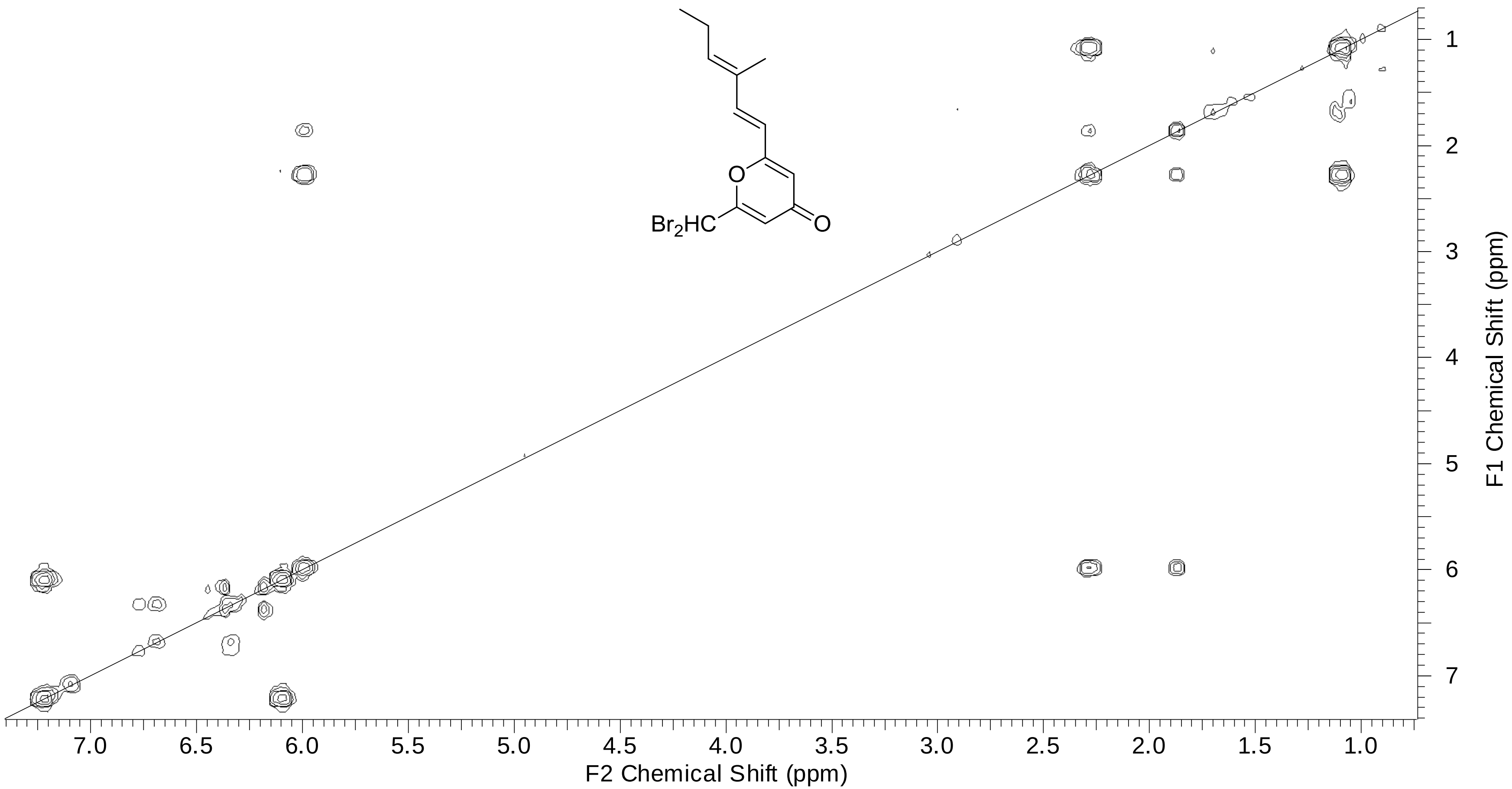

Figure S23. gHSQC (600 MHz, $CDCl_3$) of **4**.

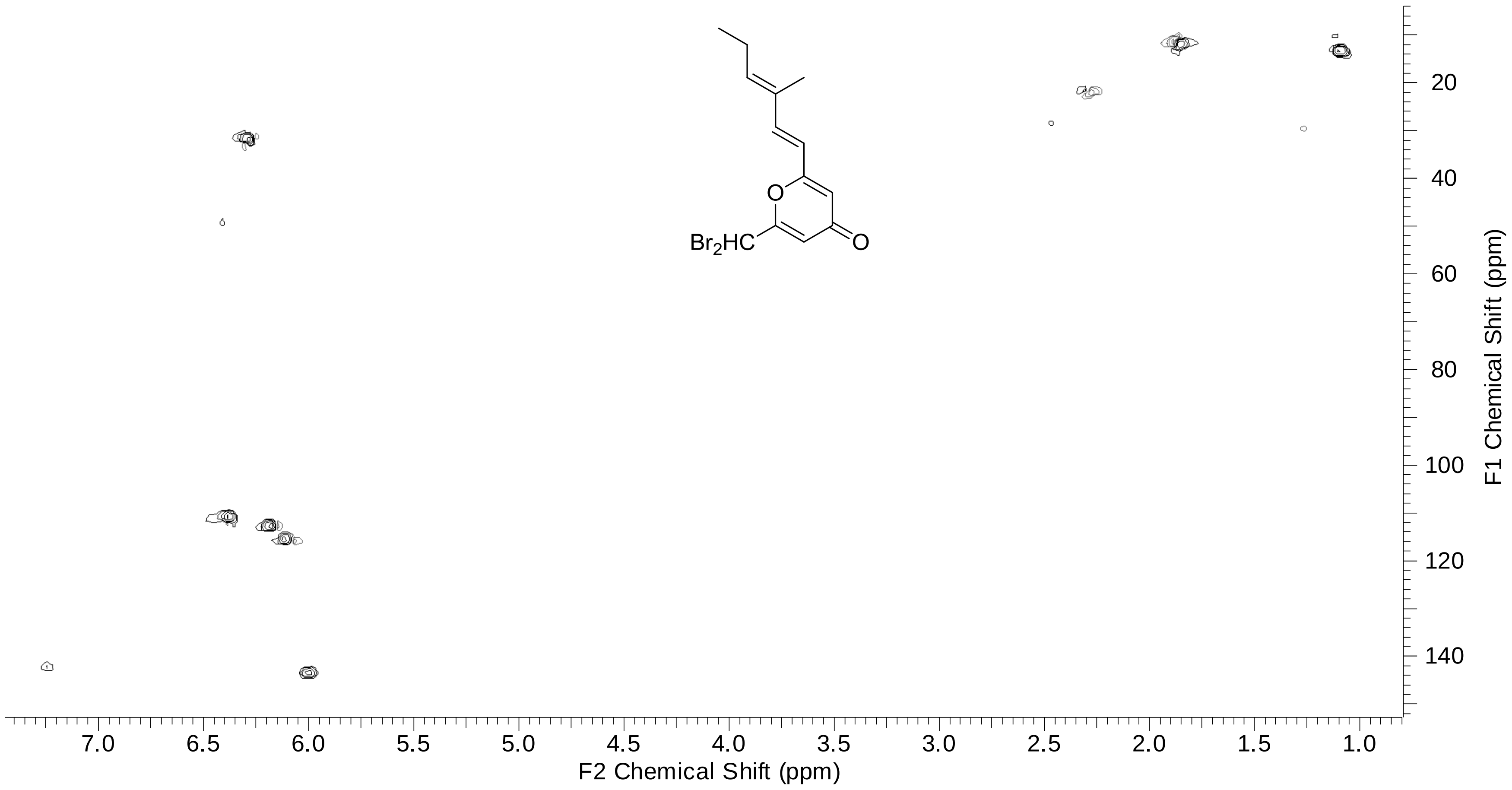

Figure S24. gHMBC (600 MHz, $CDCl_3$) of **4**.

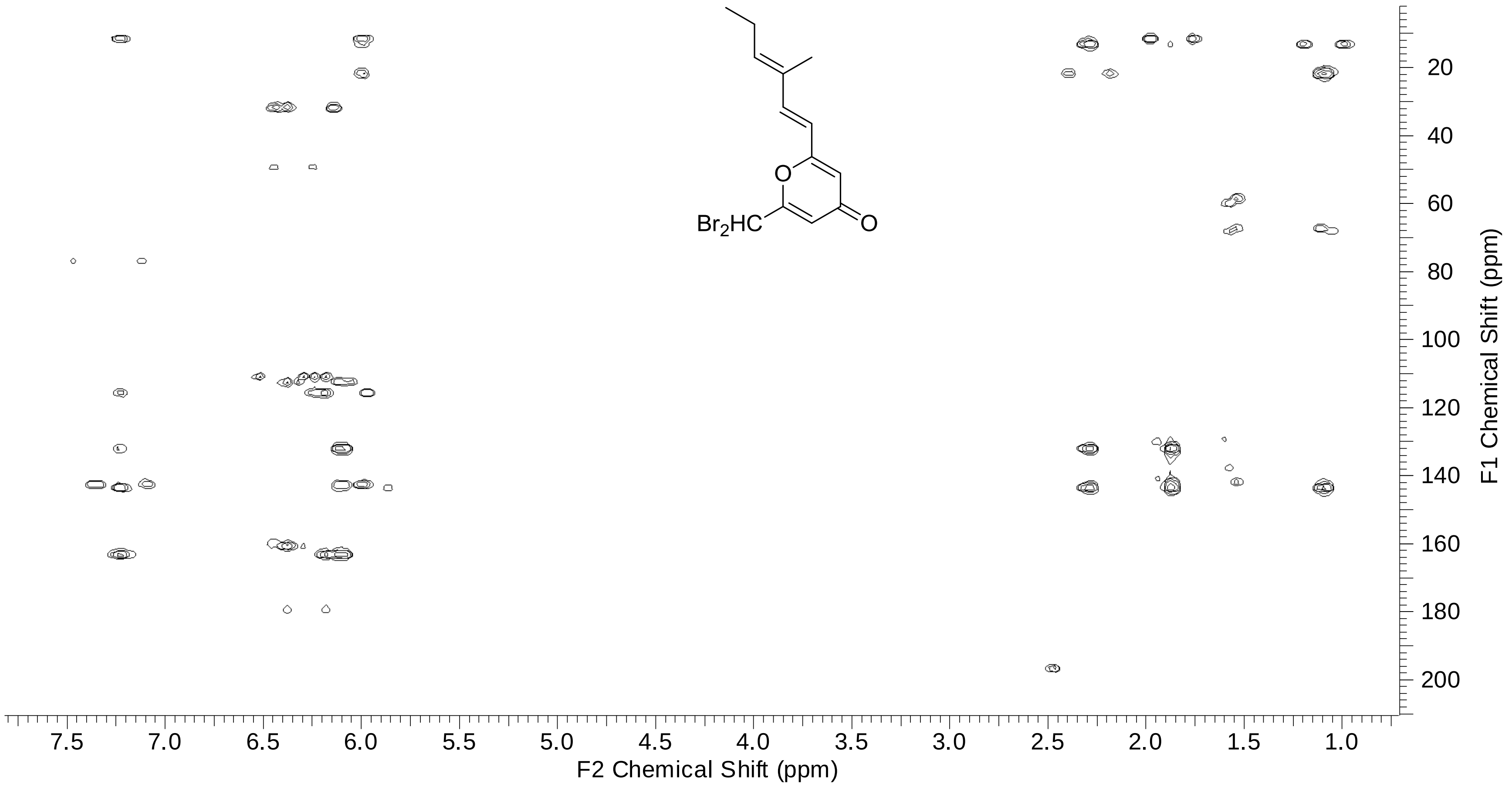

Figure S25. HRMS of **4**.

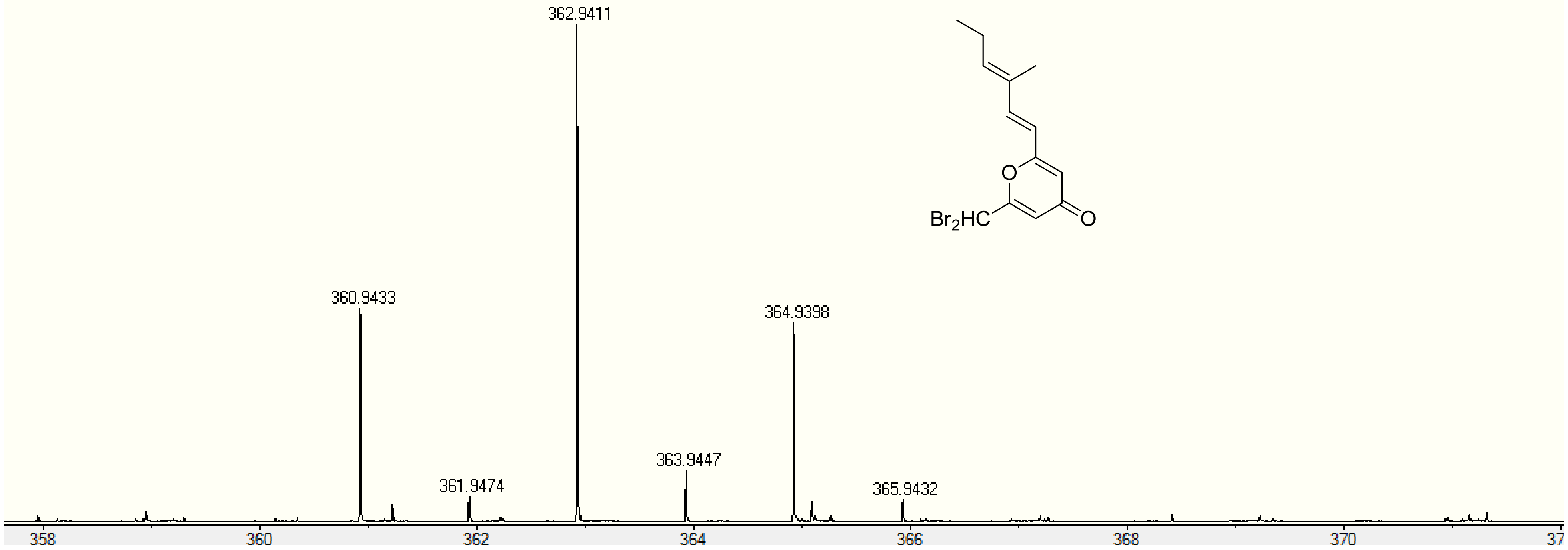

Table S1. ^{1}H and ^{13}C NMR data for **5** (600 MHz for ^{1}H, 150 MHz for ^{13}C, $CDCl_3$).

**5**			
**δ_C, mult.**	**δ_H (J in Hz)**	**COSY**	**HMBC**
13.8, CH_3	1.02, t (7.6)	2	2, 3
22.2, CH_2	2.22, qn (7.6)	1, 3	1, 3, 4
142.6, CH	5.84, t (7.3)	2	1, 2, 5, 6
132.6, C			
12.1, CH_3	1.80, s		3, 4, 6
141.3, CH	7.02, d (15.9)	7	3, 4, 5, 7, 8
116.8, CH	6.00, d (15.9)	6	3, 4, 8, 9
163.2, C			
114.0, CH	6.07, d (2.2)	11	7, 8, 10, 11
180.9, C			
112.4, CH	6.12, d (2.2)	9	9, 10, 12, 13
165.3, C			
20.8, CH	2.28, s		11, 12

Figure S26. ^{1}H NMR (600 MHz, $CDCl_3$) of **5**.

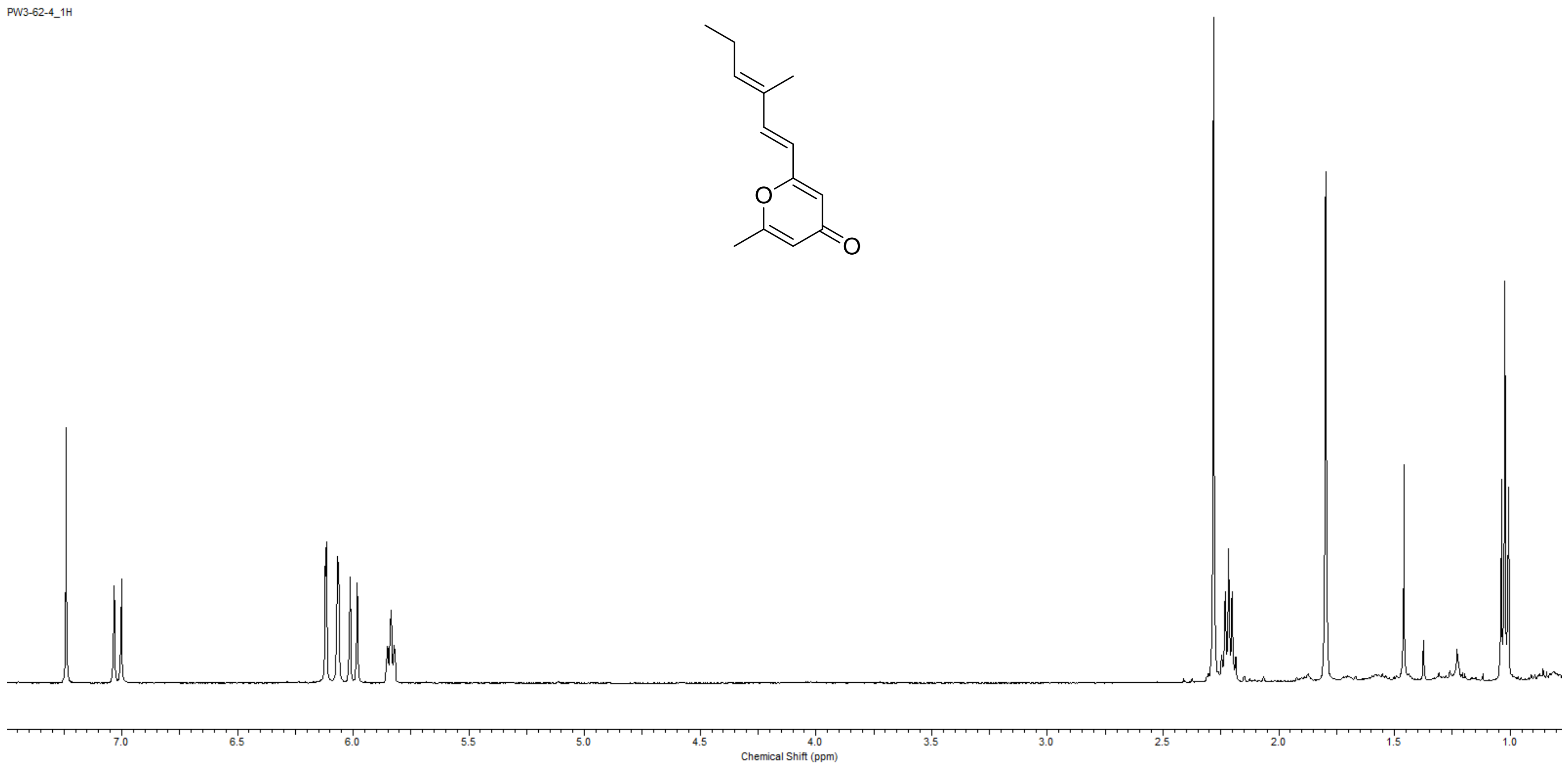

Figure S27. ^{13}C NMR (125 MHz, $CDCl_3$) of **5**.

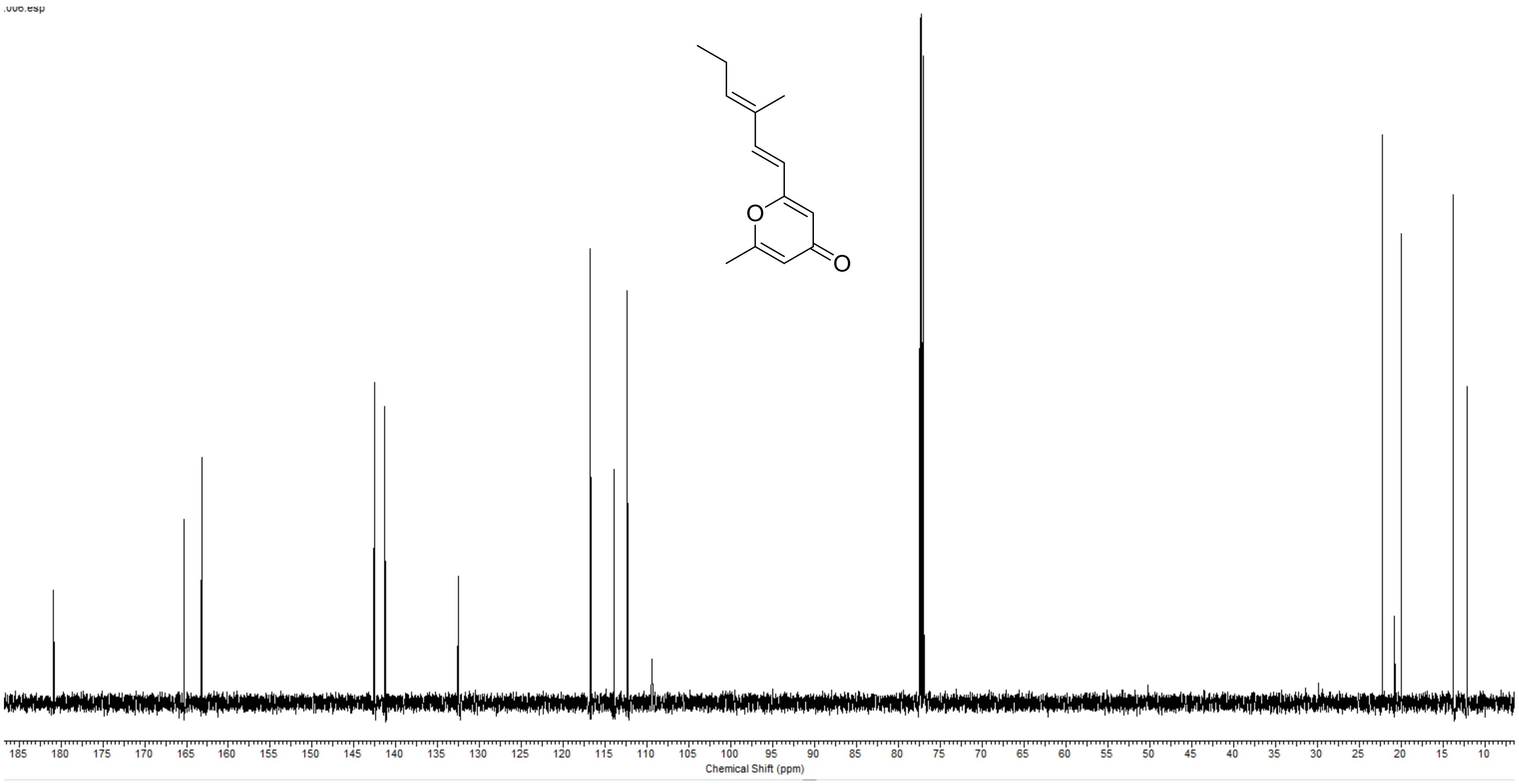

Figure S28. gCOSY (600 MHz, $CDCl_3$) of **5**.

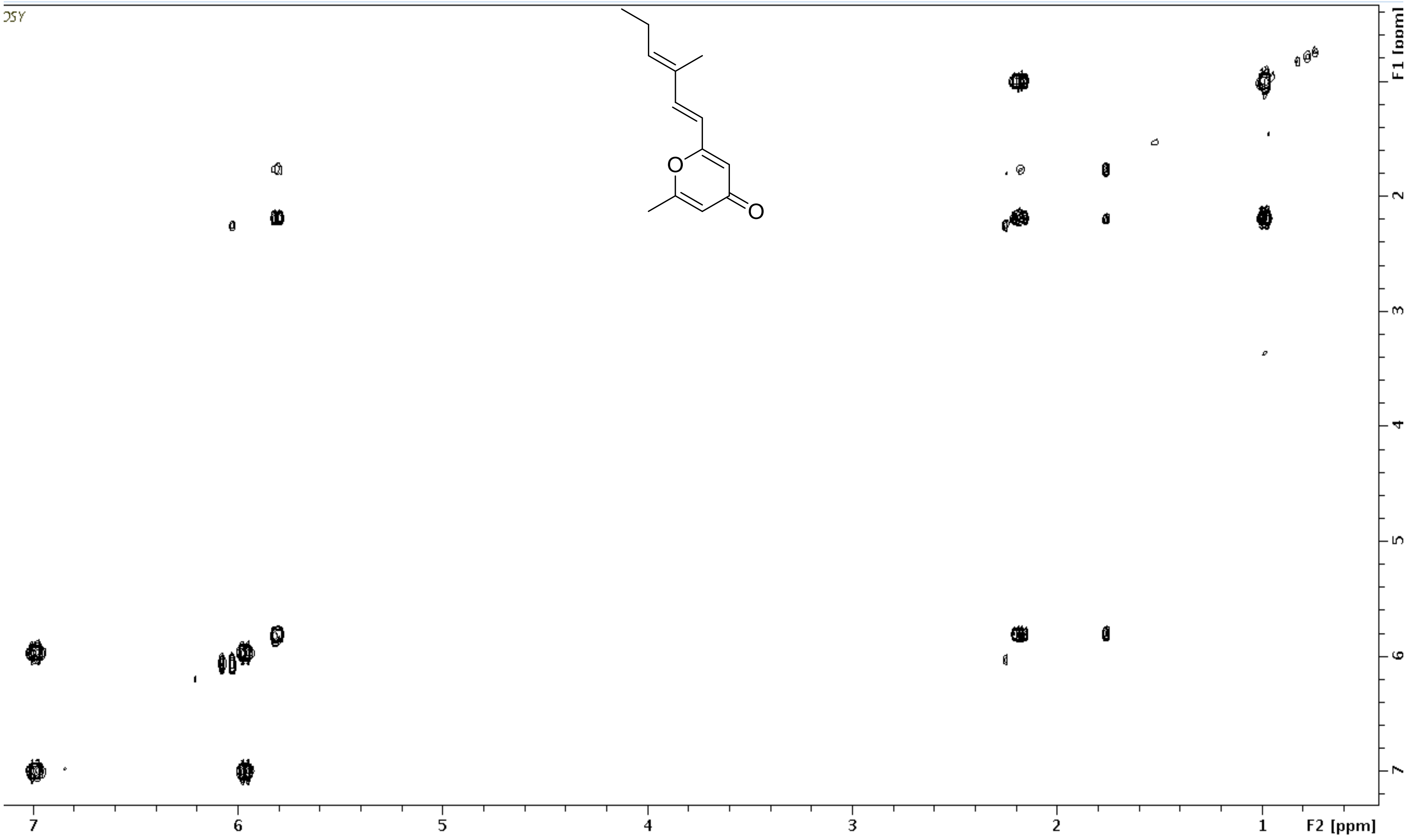

Figure S29. gHSQC (600 MHz, $CDCl_3$) of **5**.

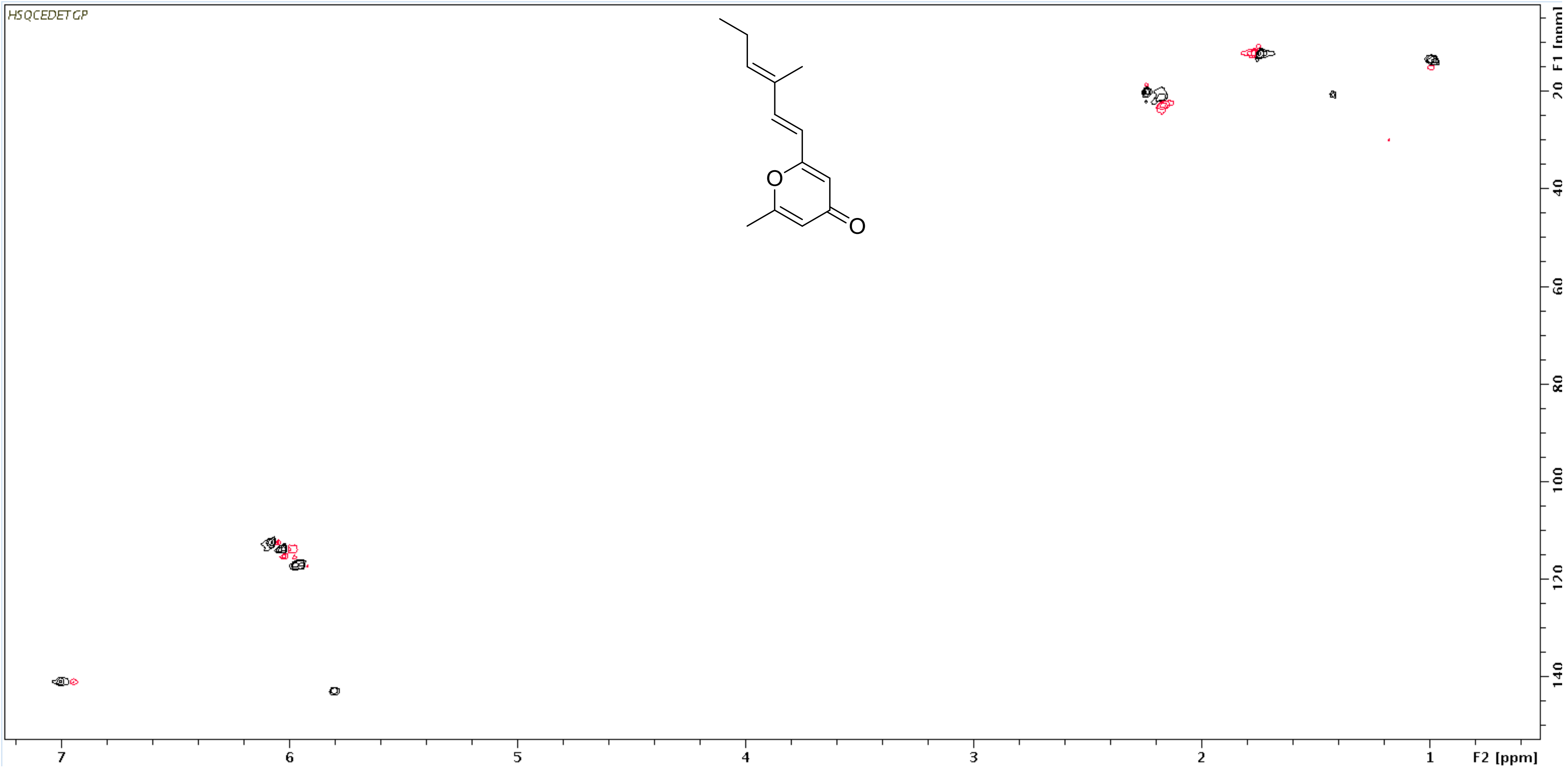

Figure S30. gHMBC (600 MHz, $CDCl_3$) of **5**.

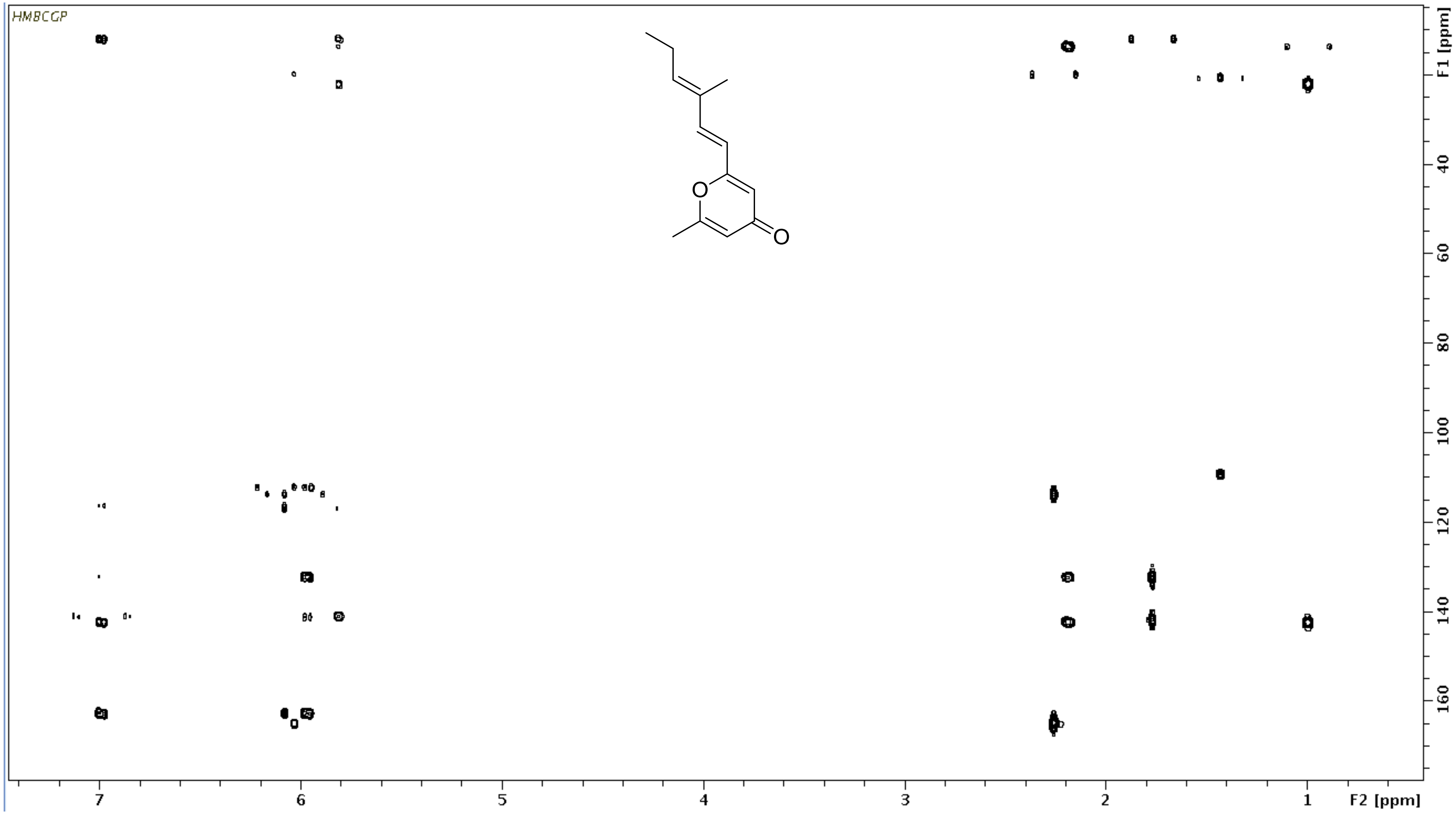

Figure S31. HRMS of **5**.

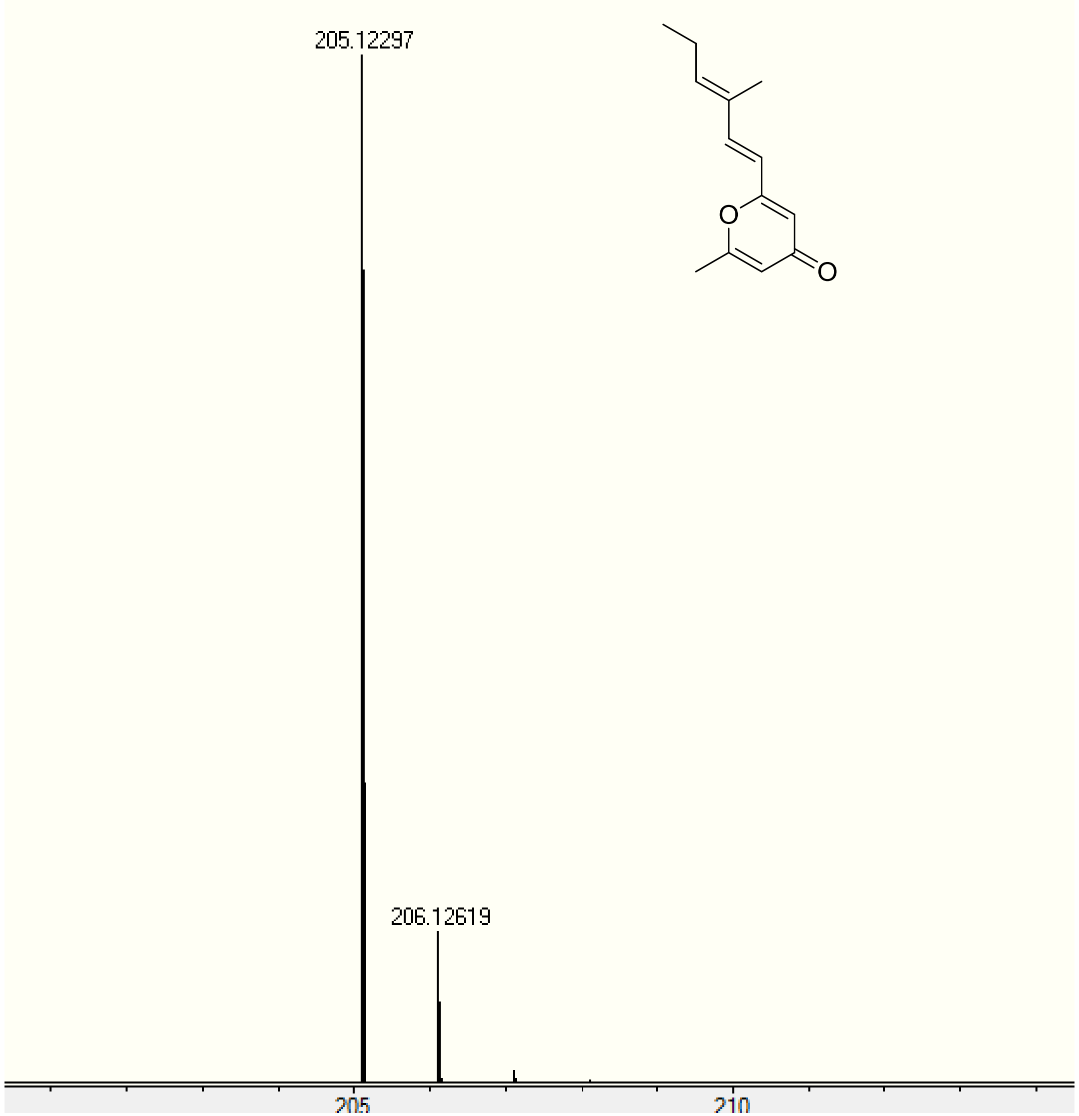

Supplement: Supplementary File 1 — Supplementary Materials (PDF, 503 KB) [file marinedrugs-11-05089-s001.pdf]
